# Comprehensive genomic profiling of upper tract urothelial carcinoma and urothelial carcinoma of the bladder identifies distinct molecular characterizations with potential implications for targeted therapy & immunotherapy

**DOI:** 10.3389/fimmu.2022.1097730

**Published:** 2023-02-03

**Authors:** Qi Tang, Wei Zuo, Chong Wan, Shengwei Xiong, Chunru Xu, Changwei Yuan, Qiangqiang Sun, Liqun Zhou, Xuesong Li

**Affiliations:** ^1^ Department of Urology, Peking University First Hospital, Beijing, China; ^2^ Precision Medicine Center, Yangtze Delta Region Institute of Tsinghua University, Jiaxing, Zhejiang, China; ^3^ Lifehealthcare Clinical Laboratory, Beijing, China

**Keywords:** UTUC (renal pelvis & ureter), UCB, targeted next-generation sequencing, genomic characterization, germline variants, DDR, mutational landscape

## Abstract

**Backgrounds:**

Despite the genomic landscape of urothelial carcinomas (UC) patients, especially those with UC of bladder (UCB), has been comprehensively delineated and associated with pathogenetic mechanisms and treatment preferences, the genomic characterization of upper tract UC (UTUC) has yet to be fully elucidated.

**Materials and methods:**

A total of 131 Chinese UTUC (74 renal pelvis & 57 ureter) and 118 UCB patients were enrolled in the present study, and targeted next-generation sequencing (NGS) of 618 cancer-associated genes were conducted to exhibit the profile of somatic and germline alterations. The COSMIC database, including 30 mutational signatures, were utilized to evaluate the mutational spectrums. Moreover, TCGA-UCB, MSKCC-UCB, and MSKCC-UTUC datasets were retrieved for preforming genomic alterations (GAs) comparison analysis between Western and Chinese UC patients.

**Results:**

In our cohort, 93.98% and 56.63% of UC patients were identified with oncogenic and actionable somatic alterations, respectively. Meanwhile, 11.24% of Chinese UC patients (of 14.50% and 7.63% of UTUC and UCB cases, respectively) were identified to harbor a total of 32 pathogenic/likely-pathogenic germline variants in 22 genes, with DNA damage repair (DDR)-associated *BRCA1* (1.20%) and *CHEK2* (1.20%) being the most prevalent. Chinese UTUC and UCB patients possessed distinct somatic genomic characteristics, especially with significantly different prevalence in *KMT2D/C/A*, *GNAQ*, *ERCC2*, *RB1*, and *PPM1D*. In addition, we also found notable differences in the prevalence of *ELF3*, *TP53*, *PMS2*, and *FAT4* between renal pelvis and ureter carcinomas. Moreover, 22.90% and 33.90% of UTUC and UCB patients, respectively, had at least one deleterious/likely deleterious alteration in DDR related genes/pathways. Subsequently, mutational signature analysis revealed that UC patients with mutational signature 22, irrespective of UTUC or UCB, consistently had the markedly higher level of tumor mutational burden (TMB), which was proved to be positively correlated with the objective complete/partial response rate in the IMvigor210 cohort. By comparison, Chinese and Western UTUC patients also differed regrading GAs in oncogenic-related genes/pathways, especially in TP53, RTK/RAS, and PI3K pathways; besides, more alterations in WNT pathway but less TP53, RTK/RAS, HIPPO, and PI3K pathways were identified in Chinese UCB.

**Discussions:**

The in-depth analysis of genomic mutational landscapes revealed distinct pathogenetic mechanisms between Chinese UTUC and UCB, and specific genomic characterizations could identify high risk population of UTUC/UCB and provided information regarding the selection of alternative therapeutic regimens.

## 1 Introduction

Urothelial carcinoma (UC), including upper tract UC (UTUC) and UC of bladder (UCB), is one of the most common genitourinary malignancies worldwide (1, 2). According to the latest cancer statistics reported by Chinese National Cancer Center, the estimated incidence and mortality rates of UCB in China have risen to 5.95/10^5^ and 2.44/10^5^, respectively (3). Among UC patients, 90%-95% tumors originate in bladder; while UTUC, only accounting for 5%-10% of UC, is arising in the renal pelvis or ureter. Due to the similar morphological and histological appearance, diagnostic and therapeutic strategies for UTUC are largely derived from those used for UCB. However, based on the data from Caucasian patients, UTUC and UCB exhibit notably distinct clinical, and molecular characteristics (4, 5). Molecular profiling is necessary to recognize the distinct pathogenetic mechanisms, and provide matched therapeutic regimens for patients with UC.

The genomic landscape of UC, including prevalent alterations in signaling pathways and driver genes, such as *TP53*, *FGFR3*, *ERBB2*, and *PIK3CA*, is closely associated with tumor development and aggressiveness, and affects treatment response (6). An earlier study discloses that *TP53* alterations are the most prevalent in UCB, simultaneously with *MLL2* (has aliases as *KMT2D*), *ARID1A* and *KDM6A* (chromatin remodeling genes) (7). Genomic alterations (GAs) comparison analysis between UTUC and UCB by a cohort study of Memorial Sloan Kettering Cancer Center (MSKCC) reveals that *TP53*, *RB1*, and *ERBB2* alterations are more frequent in UCB; whereas, *FGFR3* and *HRAS* are more prevalently altered in UTUC (8). Afterwards, the above results have been further confirmed in another Western cohort study (9). Additionally, GAs in DNA damage repair (DDR) genes are also prevalent in all UC subtypes (10), and have been demonstrated to correlate with the sensitivity to platinum-based treatments (11) and immunotherapy (12). Notably, the ingestion of aristolochic acid (AA), which can initiate aristolochic acid nephropathy (13), is markedly associated with serious kidney damage and urothelial carcinogenesis, along with a distinct mutational signature (sig) in affected UC individuals (14). Also, exposure to AA contained in some Chinese herbal medicines contributing to the damage of renal functions is highly correlated with the development of UTUC among Chinese patients (15). Recently, a pan-cancer analysis mainly based on the data from The Cancer Genome Atlas (TCGA) and International Cancer Genome Consortium (TCGA/ICGC) delineates that COSMIC (Catalogue of Somatic Mutations in Cancer) sig 22 is specifically found in tumor tissue samples with the exposure to AA, and there is an extremely high mutational burden in UC, especially in renal pelvis tumors exposed to AA. In addition, more UTUC patients have predispositions in Lynch Syndrome (LS) related features, such as tumor mutational burden (TMB) and microsatellite instability (MSI) caused by germline mutations in DNA mismatch repair (MMR) genes (16). Nonetheless, there are few studies on the pathogenic/likely-pathogenic (P/LP) germline variants of UTUC, particularly for Chinese patients.

Overall, it is essential to investigate and comprehend the germline and somatic landscape of Chinese UTUC and UCB, leveraging into substantial advances in potentiating clinical applicability, guiding clinical management, and promoting precision medicine. Currently, only small retrospective Chinese UC patient series have showed the discrepancies in the prevalence of altered genes between UTUC and UCB; however, due to the rarity of UTUC, results of these studies are limited by small sample sizes. In the present study, a total of 249 Chinese UC patient samples, including 131 UTUC and 118 UCB cases, were enrolled and performed *via* the next-generation sequencing (NGS) to characterize the genomic landscape of Chinese UC patients.

## 2 Materials and methods

### 2.1 Study samples and ethics

UC patients, comprising 131 UTUC (79 male cases & 52 female cases, and diagnosis age ranged from 36 to 86 years old) and 118 UCB (92 male cases & 26 female cases, and diagnosis age ranged from 19 to 87 years old) patient samples, were enrolled between January 1^st^, 2018 and October 9^th^, 2021, and UTUC or UCB disease was diagnosed by a qualified physician. Written informed consents of all involved patients have been collected, and this study was conducted according to the International Ethical Guidelines for Biomedical Research Involving Human Subjects, the Declaration of Helsinki, and approved by the ethics committee of Peking University First Hospital. In the present study, there was no limit regarding the gender, age, weight of involved patients when collecting UTUC or UCB patient samples; however, pregnant or breastfeeding female samples were excluded. Besides, a total of 3 qualified surgeons participated into the surgical operations and all patient samples were collected using simple randomization. Formalin-fixed, paraffin-embedded (FFPE) tumor tissues and germline DNA from matched blood samples were subsequently analyzed by using a targeted NGS platform. The representative FFPE tumor tissues were estimated by a qualified pathologist to determine whether the tumor content was sufficient (at least 20%). Of these samples, 1 UTUC & 2 UCB samples were removed because of insufficient tumor content and 1 UCB samples were lost owing to technical problems during DNA extraction, thus resulting in a total of 131 UTUC and 118 UCB samples available for subsequent analysis. Of note, all involved workers were blinded as of sample collection, processing, outcome assessment, and bioinformatic analysis.

### 2.2 DNA extraction and targeted NGS

The FFPE tumor tissue samples and white blood cell samples, including tumor genomic DNA (gDNA) and germline gDNA, respectively, were initially extracted by using QIAamp Genomic DNA Kit (Qiagen, CA, USA) according to the instructions of manufacturer. Next, the quality and quantity of purified DNA were detected and analyzed *via* using Agilent 2100 Bioanalyzer (Agilent Technologies, CA, USA) and Qubit 3.0 Fluorometer (Thermo Fisher Scientific Life Technologies, MA, USA). Then, 100 ng gDNA was sheared by a Covaris E210 system (Covaris, MA, USA), and both the FFPE tumor gDNA and germline gDNA library construction were prepared by using Accel-NGS 2S DNA Library Kit (Swift Biosciences, MI, USA). The target-enriched libraries were constructed by using xGen Lockdown Probes Kit (Integrated DNA Technology, IA, USA), and the probes were synthesized by Integrated DNA Technology, Inc for a panel of 618 genes (**Supplementary Table 1).** In the present study, bioassays were replicated three times. Eventually, paired-end sequencing with 150 bp length each read was conducted for the target-enriched libraries on the Illumina Novaseq 6000 platform (Illumina, CA, USA). The coverage of at least 1000× and 200× was achieved for tissue and germline gDNA, respectively.

### 2.3 Sequencing data analysis and variant interpretation

The FASTQ files of paired-end sequencing reads were obtained by the software FASTP (FASTP, RRID : SCR_016962, https://github.com/OpenGene/fastp), and the quality control was also conducted *via* CASAVA (CASAVA, RRID : SCR_001802, http://support.illumina.com/sequencing/sequencing_software/casava.html). The raw sequencing data were then aligned to the UCSC Human Genome Reference hg19 by Burrows-Wheeler alignment tool and a binary sequence alignment map (BAM) file was generated. The duplicate removal and local realignment were performed by using Picard (Picard, RRID : SCR_006525, http://broadinstitute.github.io/picard/) to improve the mapping quality. Variant calling (single nucleotide variation (SNV), small insertions/deletions (indels), etc.) was based on the Genome Analysis Toolkit (GATK, RRID : SCR_001876, https://software.broadinstitute.org/gatk/download/), and they were annotated by using the software ANNOVAR (ANNOVAR, RRID : SCR_012821, http://www.openbioinformatics.org/annovar/). In addition, TMB was defined as the number of identified variants per megabase (Mb). The somatic alterations were interpretated according to the standards and guidelines of Association for Molecular Pathology, American Society of Clinical Oncology, and College of American Pathologists. The germline variants were determined by a molecular geneticist in accordance with the standards and guidelines of American College of Medical Genetics and Genomics, and the Association for Molecular Pathology, it is necessary to state that variants with unknown significance were also evaluated but not reported in the present study. The oncogenic alterations were identified in ClinVar (ClinVar, RRID : SCR_006169, http://www.clinvar.com/). Actionable GAs analysis was conducted in OncoKB (OncoKB, RRID : SCR_014782, https://www.oncokb.org/).

### 2.4 DDR pathways, mutational signature and alterations analysis

Thirty-four DDR genes (**Supplementary Table 2**), included in six canonical pathways of Checkpoint, Fanconi Anemia (FA), Homologous Recombination (HR), Mismatch Repair (MMR), Nucleotide Excision Repair (NER), and others, were obtained as previously described (11), which was utilized for the analysis of somatic alteration profiling. Besides, an additional *EPCAM* was usually used to characterize potential UC patients with LS as a MMR-associated gene (17) during the investigation of germline variants in our cohort. Deleterious alterations were determined according to the interpretation of OncoKB database *via* cBioPortal (cBioPortal, RRID : SCR_014555, http://www.cbioportal.org/).

The landscape of GAs of UC patients was exhibited in the Oncoprint plots by using the package maftools. According to the previously described oncogenic signaling pathways in 33 cancer types (18), somatic GAs in our study were categorized into following canonical pathways: TP53, RTK/RAS, DDR, NOTCH, PI3K, Cell Cycle, WNT, HIPPO, TGF-, MYC, and NRF2. Besides, the comparison of GA types of selected genes between UTUC and UCB was visualized in the lollipop plots also by using the package maftools. In addition, 30 COSMIC mutational signatures were downloaded from the COSMIC website (COSMIC, RRID : SCR_002260, http://cancer.sanger.ac.uk/cancergenome/projects/cosmic/), and mutational signature analysis was conducted by using nonnegative matrix factorization from the 96-channel mutational profiles (19).

### 2.5 Genomic data of western UC patients

Eventually, clinical and genomic data of UTUC (MSK, Nat. Commun. 2020) (20), namely MSKCC-UTUC cohort in the present study, were downloaded to investigate the difference in GAs between Chinese and Western UTUC patients, and the clinical comparison analysis was shown in **Supplementary Table 3**. In addition, clinical and genomic data of UCB from TCGA, PanCancer Atlas (21) and MSKCC, Eur Urol 2014 (22), namely TCGA-UCB and MSKCC-UCB cohort, respectively, were retrieved to investigate the difference in GAs between Chinese and Western UCB patients, and the clinical comparison analysis between Chinese and TCGA-UCB or MSKCC-UCB cohorts was exhibited in **Supplementary Table 4, 5**. In the present study, muscle-invasive bladder cancer (MIBC) patients were screened out for subsequent analysis. It should be mentioned that a shared panel of 209 genes (**Supplementary Table 6**) was used when comparing GAs between Chinese UTUC and MSKCC-UTUC cohorts, and also the same shared panel of 209 genes was used when comparing GAs between Chinese UCB and MSKCC-UCB cohorts, besides, a panel of 618 genes (**Supplementary Table 1**) was used to filter GAs of UCB patients from the TCGA-UCB cohort for subsequent analysis. The IMvigor210 cohort (23), including UC patients treated with immunotherapy by anti-PD-L1 therapy, was downloaded to explore the relationship between TMB level and immunotherapy response. The defined criteria of therapy response: CR: complete response, PR: partial response, SD: stable disease, PD: progressive disease.

### 2.6 Statistical analysis

Statistical analysis was conducted in R studio (v.3.4.3, https://rstudio.com/) or by using the software Statistical Package for the Social Sciences (SPSS, RRID : SCR_002865, http://www-01.ibm.com/software/uk/analytics/spss/) and GraphPad Prism v8.0 (GraphPad Prism, RRID : SCR_002798, http://www.graphpad.com/), and chi-square and Fisher’s exact tests were used to determine the statistical significance of differences among demographic categorical variables, and the Kruskal-Wallis’ test was used to compare the continuous variables. The p-value < 0.05 was considered statistically significant.

## 3 Results

### 3.1 Patient characteristics

After investigation, all involved Chinese UC samples (**Table 1**), including 131 UTUC and 118 UCB tumors (UCB samples had no prior history of UTUC), were found with valid somatic alterations, and the median and mean TMB values were 12.00 [0.66, 277.32] and 17.64, respectively. Besides, the median and mean TMB values of UTUC vs. UCB were 9.88 vs. 14.00 and 17.31 vs. 18.00, respectively. Except a significantly higher proportion of female patients diagnosed with UTUC tumors instead of UCB, no statistically significant difference in other clinical features was observed between UTUC and UCB in our cohort (**Table 1**).

**Table 1:** UTUC and UCB patient characteristics in Chinese cohort.

Variables	UTUC (N = 131)	UCB (N = 118)	p-value
Diagnosis age	66 [36, 86]	66 [19, 87]	0.59
Gender			
Male	79	92	**< 0.01**
Female	52	26	
Smoker			
Yes	20	25	0.28
No	23	17	
NA	88	76	
Tumor site			
Renal pelvis	74	NA	NA
Ureter	57	NA	
Clinical stage			
I/II	47	32	0.17
III/IV	84	84	
NA	0	2	

UTUC, upper tract urothelial carcinoma; UCB, urothelial carcinoma of the bladder; TMB, tumor mutation burden; Bold represented there was a statistical significance; NA, not applicable.

### 3.2 Somatic and actionable alterations of Chinese UC patients

**Figure 1:**
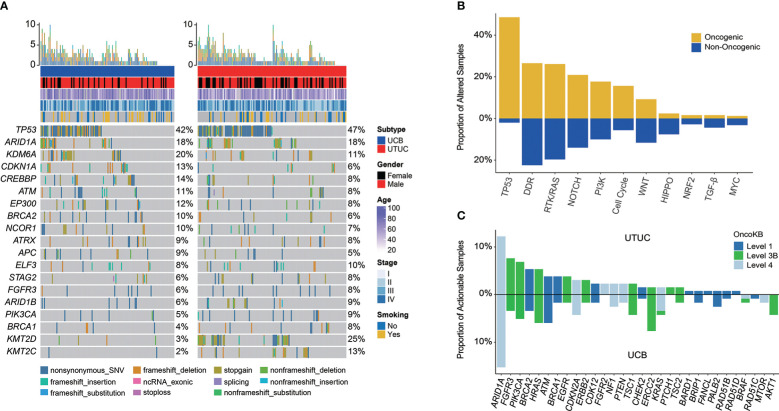
Profiling of prevalent somatic and actionable alterations in Chinese UTUC and UCB. **(A)** Comparison of the most prevalently altered genes between Chinese UTUC and UCB. **(B)** Oncogenic canonical pathways in Chinese UC. **(C)** Comparison of actionable alterations between Chinese UTUC and UCB. UTUC, upper tract urothelial carcinoma, UCB, urothelial carcinoma of the bladder.

In total, genomic landscape of Chinese UC samples demonstrated that the most frequently altered genes (**Figure 1A**) included *TP53* (44.58%), *ARID1A* (18.00%), and *KDM6A* (15.26%); whereas, it was noticed that the alteration frequencies of *FGFR3* were only 7.63% and 5.93%, respectively in UTUC and UCB. When grouping GAs by functional significance, it was revealed that the top prevalently enriched pathways were TP53 (50.60%), DDR (49.00%), RTK/RAS (45.78%), NOTCH (34.94%), PI3K (27.71%), and Cell Cycle (21.29%). While 93.98% (234/249) of Chinese UC patients were identified as having oncogenic alterations, which were the most abundantly enriched in TP53 signaling pathway (48.59%, **Figure 1B**).

Furthermore, a total of 141 (56.63%) Chinese UC patients with 220 actionable alterations were identified in our cohort. Irrespective of UTUC or UCB, it was found that more patients had level 3 actionable GAs (level 3 vs. level 4 vs. level 1, UTUC: 44.44% vs. 32.22% vs. 23.33%; UCB: 39.33% vs. 37.08% vs. 23.60%, **Supplementary Figure 1**). UC patients had the most actionable GAs in *ARID1A* (N = 34, 13.65%; UTUC vs. UCB: 12.21% vs. 15.25%). Besides, remaining actionable GAs were mostly enriched in the RTK/RAS, PI3K, and DDR related signaling pathways (**Figure 1C**). According to the statistical analysis, there was no difference in the prevalence of actionable GAs between Chinese UTUC and UCB, except that more *AKT1* and *ERCC2* actionable GAs were presented in Chinese UCB (p < 0.05, **Figure 1C**). In comparison with the TCGA-UCB cohort, more *AKT1* (4.24% vs. 0.24%), *EGFR* (1.69% vs. 0.00%), and *PALB2* (2.54% vs. 0.24%) actionable GAs occurred in the Chinese UCB cohort; whereas, *ERBB2* (9.29% vs. 1.69%), *FGFR3* (12.96% vs. 3.39%), *KDM6A* (20.29% vs. 0.00%), and *PIK3CA* (18.34% vs. 5.08%) actionable GAs were prevalent in the TCGA-UCB cohort (p < 0.05, **Supplementary Figure 2**). Regarding the comparison of actionable GAs between Chinese UCB and MSKCC-UCB cohort, it was discovered that MSKCC-UCB cohort had more prevalent actionable GAs in *ARID1A* (27.52% vs. 15.25%), *FGFR3* (18.35% vs. 3.39%), *KDM6A* (37.61% vs. 0.00%), and *PIK3CA* (22.02% vs. 5.08%), while *ERCC2* (7.63% vs. 0.92%) actionable GAs were more prevalent in the Chinese UCB cohort (p < 0.05, **Supplementary Figure 3**). In addition, *FGFR3* (44.34% vs. 8.47%), *KDM6A* (23.48% vs. 0.00%), and *TSC1* (10.43% vs. 2.54%) actionable GAs were more frequent in the MSKCC-UTUC cohort (p < 0.05, **Supplementary Figure 4**)

### 3.3 Spectrum of germline variants in Chinese UC patients

**Figure 2:**
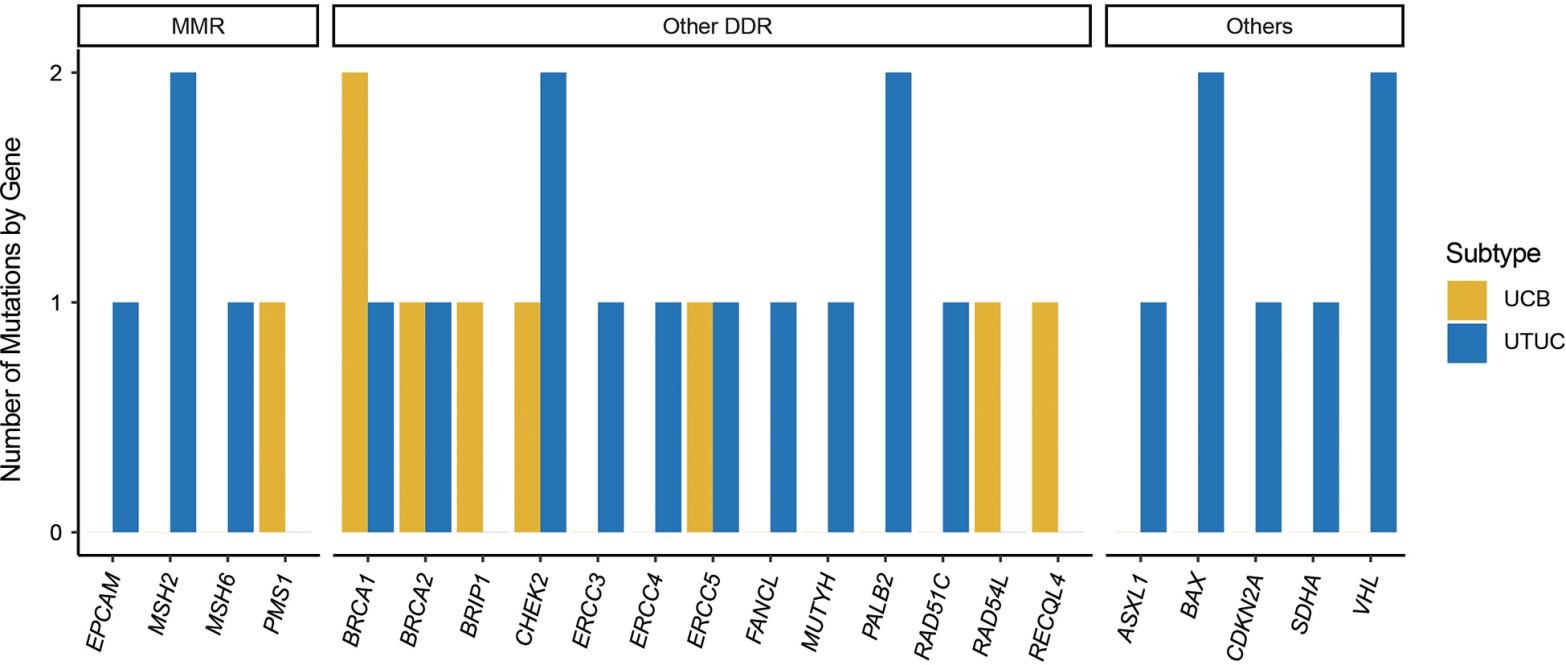
Germline pathogenic/likely-pathogenic variants in Chinese UTUC and UCB. UTUC, upper tract urothelial carcinoma, UCB, urothelial carcinoma of the bladder; MMR, mismatch repair; DDR, DNA damage repair.

In addition, a total of 32 P/LP germline variants were identified within 11.24% (28/249) of individuals with UC in our cohort, including 14.50% and 7.63% of UTUC and UCB patients (**Table 2**). Interestingly, there were four UTUC patients harboring two P/LP germline variants. The top prevalently altered genes in UC were *BRCA1* (N = 3, 1.20%, UCB vs UTUC: 2:1), *CHEK2* (N = 3, 1.20%, UCB vs UTUC: 1:2), *MSH2* (N = 2, 0.80%, UCB vs UTUC: 0:2), *ERCC5* (N = 2, 0.80%, UCB vs UTUC: 1:1), *BRCA2* (N = 2, 0.80%, UCB vs UTUC: 1:1), *BAX* (N = 2, 0.80%, UCB vs UTUC: 0:2), and *PALB2* (N = 2, 0.80%, UCB vs UTUC: 0:2). In addition, 25 out of 32 P/LP germline variants were in genes associated with DDR in UC, of which 5 were in MMR-associated genes, including *MSH2* (N = 2), *MSH6* (N = 1), *PMS1* (N = 1), *EPCAM* (N = 1). In UCB, it was observed that the Chinese UCB cohort had the germline variants of MMR-associated *PMS1* (N = 1), and other DDR-associated *BRCA1* (N = 2), *BRCA2* (N = 1), *BRIP1* (N = 1), *CHEK2* (N = 1), *ERCC5* (N = 1), *RAD54L* (N = 1), & *RECQL4* (N = 1). Whereas, the Chinese UTUC cohort harbored the germline variants of MMR-associated *MSH2* (N = 2), *MSH6* (N = 1), *EPCAM* (N = 1), and other DDR-associated *BRCA1* (N = 1), *BRCA2* (N = 1), *CHEK2* (N = 2), *ERCC3* (N = 1), *ERCC4* (N = 1), *ERCC5* (N = 1), *FANCL* (N = 1), *MUTYH* (N = 1), *PALB2* (N = 2), *RAD51C* (N = 1), and non-DDR-associated *ASXL1* (N = 1), *BAX* (N = 2), *CDKN2A* (N = 1), *SDHA* (N = 1) & *VHL* (N = 2) (**Figure 2**).

**Table 2:** The detail information of 32 germline P/PL variants in the Chinese cohort.

Patient	Age	Gender	Stage	Gene	Chr.	Exon	Coding sequence change	Amino acid change
UTUC								
**1**	69	Male	IV	*MUTYH*	1	10	c.C790T	p.Q264X
				*PALB2*	16	9	c.G2968T	p.E990X
**2**	72	Male	III	*CDKN2A*	9	2	c.C364T	p.R122X
				*MSH2*	2	12	c.C1861T	p.R621X
**3**	57	Female	III	*BAX*	19	3	c.C386A	p.S129X
				*ERCC4*	16	8	c.1441_1444del	p.K481fs
**4**	83	Female	II	*VHL*	3	1	c.115dupG	p.S38fs
				*VHL*	3	1	c.120_126del	p.P40fs
5	36	Male	III	*SDHA*	5	1	c.A1G	p.M1V
6	51	Male	III	*BRCA1*	17	10	c.3407delC	p.P1136fs
7	54	Male	III	*ERCC5*	12	23	c.4753delA	p.N1585fs
8	54	Male	III	*FANCL*	2	13	c.1066_1067del	p.S356fs
9	57	Male	IV	*EPCAM*	2	NA	c.344_352delTGTGCTGGT insCGTGCTG	p.Met115ThrfsTer17
10	60	Male	IV	*CHEK2*	22	11	c.C1111T	p.H371Y
11	69	Male	IV	*PALB2*	16	5	c.2192dupT	p.L731fs
12	76	Male	III	*MSH6*	2	1	c.C194A	p.S65X
13	83	Male	II	*ASXL1*	20	11	c.C1564T	p.Q522X
14	33	Female	IV	*BRCA2*	13	20	c.8529_8530del	p.N2843fs
15	39	Female	IV	*MSH2*	2	13	c.C2038T	p.R680X
16	56	Female	IV	*ERCC3*	2	1	c.A1G	p.M1V
17	64	Female	IV	*RAD51C*	17	8	c.1022_1026del	p.I341fs
18	72	Female	IV	*BAX*	19	4	c.260dupC	p.S87fs
19	73	Female	IV	*CHEK2*	22	11	c.C1111T	p.H371Y
UCB								
20	42	Male	III	*BRIP1*	17	17	c.C2392T	p.R798X
21	50	Male	IV	*PMS1*	2	3	c.C163T	p.R55X
22	59	Male	III	*BRCA1*	17	23	c.5470_5477del	p.I1824fs
23	62	Male	IV	*BRCA2*	13	11	c.G5416T	p.E1806X
24	63	Male	IV	*BRCA1*	17	10	c.T938G	p.L313X
25	66	Male	IV	*ERCC5*	13	19	c.3821_3827del	p.H1274fs
26	67	Male	IV	*RAD54L*	1	19	c.2081_2082del	p.P694fs
27	67	Male	IV	*RECQL4*	8	21	c.3430delC	p.R1144fs
28	63	Female	II	*CHEK2*	22	11	c.C1111T	p.H371Y

Chr, chromosome; UTUC, upper tract urothelial carcinoma; UCB, urothelial carcinoma of the bladder; Bold, patients with two germline P/PL variants; NA, not applicable.

### 3.4 Comparison of the genomic difference between Chinese UTUC and UCB

**Figure 3:**
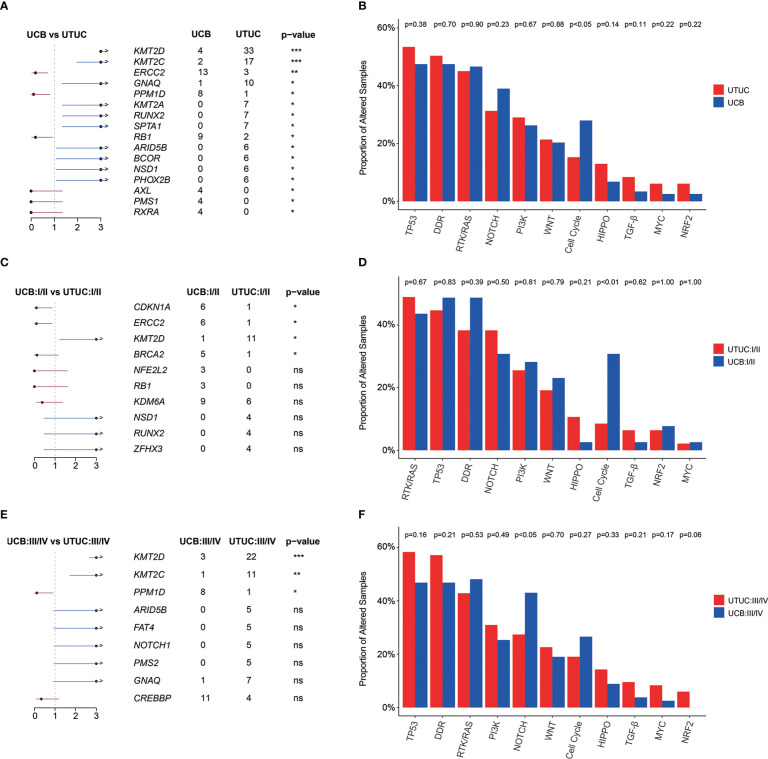
Genomic differences between Chinese UTUC and UCB. **(A)** Prevalently altered genes, respectively in UTUC and UCB. **(B)** Comparison of the differences in the prevalence of commonly altered molecular pathways between UTUC and UCB. **(C)** Prevalently altered genes at early stage, respectively in UTUC and UCB. **(D)** Comparison of the differences in the prevalence of commonly altered molecular pathways at early stage, between UTUC and UCB. **(E)** Prevalently altered genes at advanced stage, respectively in UTUC and UCB. **(F)** Comparison of the differences in the prevalence of commonly altered molecular pathways at advanced stage, between UTUC and UCB. UTUC, upper tract urothelial carcinoma; UCB, urothelial carcinoma of the bladder. *: p < 0.05; **: p < 0.01; ****: p < 0.0001. ns, not significant.

GA enrichment analysis revealed that *KMT2D* (25.19% vs. 3.39%), *KMT2C* (12.98% vs. 1.69%), *GNAQ* (7.63% vs. 0.85%), *KMT2A* (5.34% vs. 0.00%), *RUNX2* (5. 34% vs. 0.00%), and *SPTA1* (5.34% vs. 0.00%) were significantly more prevalently altered in UTUC; whereas, *ERCC2* (11.02% vs. 2.29%), *RB1* (7.63% vs. 1.53%), and *PPM1D* (6.78% vs. 0.76%) were more prevalently altered in UCB instead (**Figure 3A**). Grouped by signaling pathway, it was found that there was no significant difference in the prevalence of altered pathway between UTUC and UCB tumors, with the exception of Cell Cycle signaling pathway which was more prevalent in UCB (27.97% vs. 15.27%, **Figure 3B**).

The difference in the prevalence of *KMT2D* between UTUC and UCB was consistent in both the diseases with early (I/II) and advanced (III/IV) clinical stages. Meanwhile, some novel difference emerged, such as more *ERCC2* (18.75% vs. 2.13%), *CDKN1A* (18.75% vs. 2.13%), *BRCA2* (15.63 vs. 2.13%) and Cell Cycle signaling pathway alterations (37.50% vs. 8.51%) were prevalent in early stage UCB (**Figures 3C, D**). For tumors in advanced clinical stage (III/IV), *KMT2C* (13.10% vs. 1.19%) and *PPM1D* (1.19% vs. 9.52%) showed significantly differed prevalence in UTUC and UCB (**Figure 3E**). More prevalent alterations in the NRF2 pathway (p = 0.06) but less alterations in NOTCH pathways (p < 0.05) were identified in patients with advanced UTUC, comparing with those with advanced UCB (**Figure 3F**). Additionally, identification of *FGFR3* alteration site further revealed that p.Arg248Cys (0.00% vs. 10.64%, p < 0.05, **Supplementary Figure 5**) was less prevalent in early-stage UCB when compared with early-stage UTUC.

### 3.5 Genomic landscape, stratified by clinical stage, of Chinese UTUC & UCB

**Figure 4:**
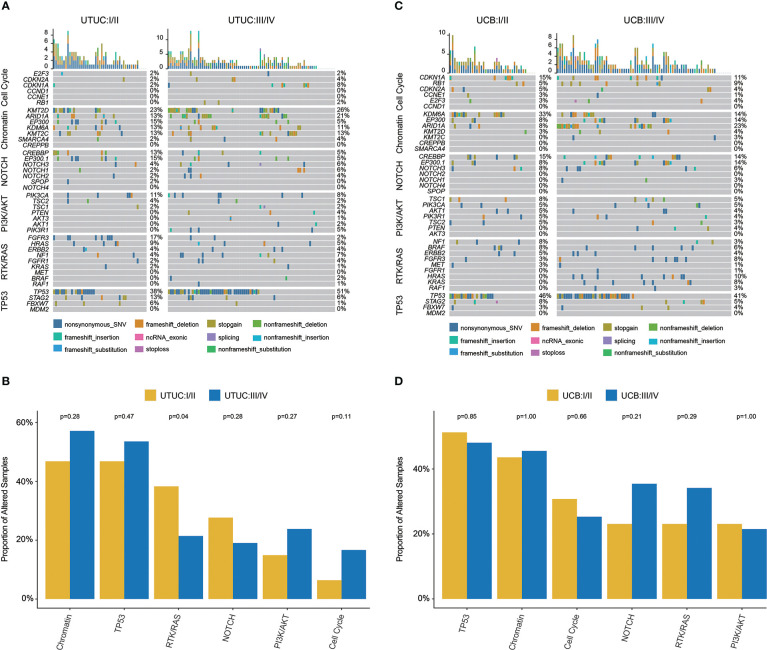
Genomic landscape, stratified by clinical stages and molecular pathways, of Chinese UTUC and UCB. **(A)** Differences of prevalent GAs between early-stage and advanced tumors in UTUC. **(B)** Comparison of the differences in the prevalence of commonly altered molecular pathways between early-stage and advanced tumors in UTUC. **(C)** Differences of prevalent GAs between early-stage and advanced tumors in UCB. **(D)** Comparison of the differences in the prevalence of commonly altered molecular pathways between early-stage and advanced tumors in UCB. UTUC, upper tract urothelial carcinoma; UCB, urothelial carcinoma of the bladder.

In UTUC, the most prevalently altered genes were *TP53* (38%), *KMT2D* (23%), and *FGFR3* (17%) in clinical stage I/II tumors, while *TP53* (51%), *KMT2D* (26%), and *ARID1A* (21%) in clinical stage III/IV tumors (**Figure 4A**). Of note, the frequency of *FGFR3* (17.02% vs. 2.38%) and *EP300* (14.89% vs. 4.76%) was significantly higher in UTUC patients with low clinical stage, along with a higher prevalence of GAs in RTK/RAS pathway (38.30% vs. 21.43%, **Figure 4B**).

For UCB, *TP53* (46%), *KDM6A* (33%), and *CDKN1A* (15%) were the most prevalent in clinical stage I/II tumors; whereas, *TP53* (41%), *ARID1A* (23%), and *EP300* (14%) were the most prevalent in clinical stage III/IV tumors (**Figure 4C**). In addition, it was found that *KDM6A* (34.38% vs. 14.28%) was more prevalent in UCB tumors with lower clinical stage, but *HRAS* (9.52% vs. 0.00%) was more prevalent in UCB tumors with advanced clinical stage. While there was no difference in the frequency of altered signaling pathways between lower and higher clinical stage UCB tumors (**Figure 4D**).

### 3.6 Genomic comparison between renal pelvis and ureter tumor

**Figure 5:**
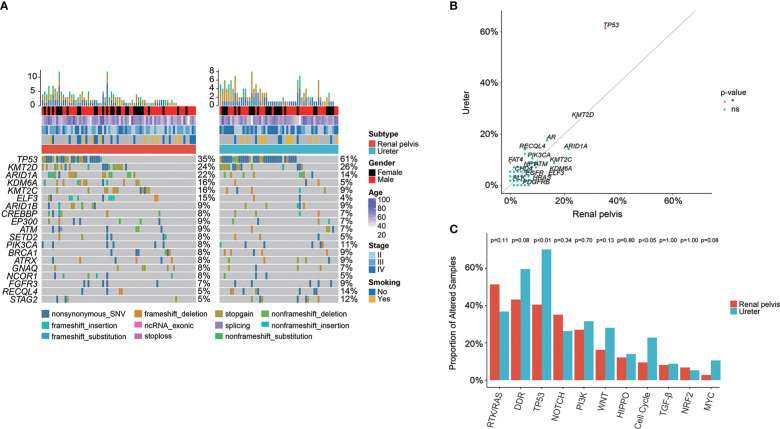
Genomic differences between renal pelvis and ureter tumors in the Chinese cohort. **(A)** Comparison of the most prevalently altered genes between renal pelvis and ureter. **(B)** Enrichment of GAs for renal pelvis and ureter tumors. **(C)** Comparison of the differences in the prevalence of commonly altered molecular pathways between renal pelvis and ureter. *: p < 0.05. ns, not significant.

To fully understand the genomic profile of Chinese UTUC patients, we further compared the genomic difference between UTUC patients with different disease sites. The oncoprint plot (**Figure 5A**) initially exhibited the genomic landscape of Chinese UTUC samples including 74 renal pelvis & 57 ureter tumors (**Table 3**). GA enrichment analysis based on the primary tumor location (renal pelvis vs. ureter) revealed that *ELF3* GAs (14.86% vs. 3.51%) were more enriched in renal pelvis tumors; conversely, the frequency of *TP53* (61.40% vs. 35.14%), *PMS2* (8.77% vs. 0.00%), *FAT4* (8.77% vs. 0.00%) was significantly higher in patients with ureter tumors (**Figure 5B**). Correspondingly, significantly more GAs in TP53 (70.18% vs. 40.54%) and Cell Cycle (22.81% vs. 9.46%) signaling pathways were observed within patients with ureter tumors, compared with those with renal pelvis tumors (**Figure 5C**).

**Table 3:** UTUC patient (Renal pelvis & Ureter) characteristics in Chinese cohort.

Variables	Renal pelvis (N = 74)	Ureter (N = 57)	p-value
**Diagnosis age**	64 [36, 84]	66 [36, 86]	0.34
Gender			
Male	46	33	0.72
Female	28	24	
Smoker			
Yes	9	11	0.07
No	17	6	
NA	48	40	
Clinical stage			
I/II	28	19	0.71
III/IV	46	38	

UTUC, upper tract urothelial carcinoma; NA, not applicable.

### 3.7 Alterations in DDR related genes/pathways of Chinese UTUC & UCB

**Figure 6:**
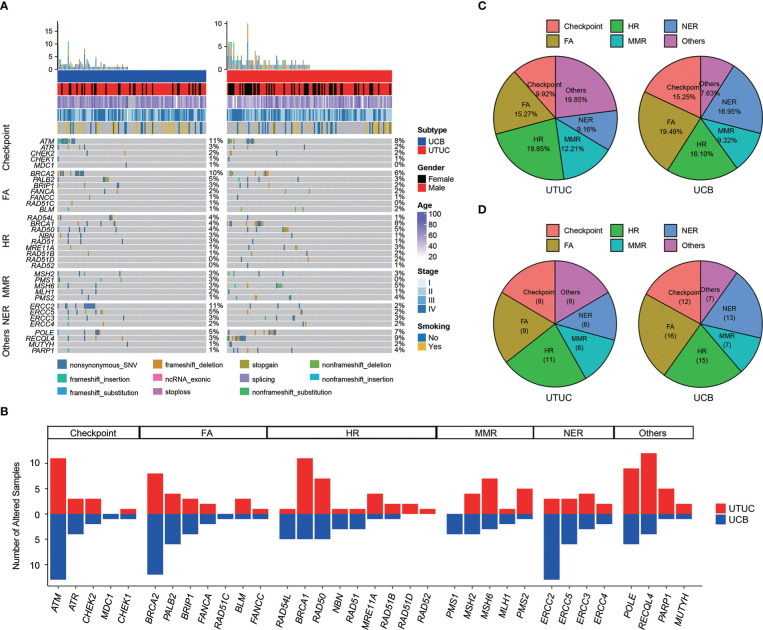
Differences in the prevalence of DDR related genes/pathways between Chinese UTUC and UCB. **(A)** Comparison of prevalent alterations in thirty-four DDR related genes between Chinese UTUC and UCB. **(B)** Number of UTUC and UCB patients harboring at least one alteration in six DDR-related canonical pathways of Checkpoint, FA, HR, MMR, NER, and Others. **(C)** Comparison of the differences in the prevalence of altered DDR-related pathways between UTUC and UCB. **(D)** Comparison of the differences in the prevalence of deleterious/likely deleterious alterations in DDR related pathways between UTUC and UCB. UTUC, upper tract urothelial carcinoma; UCB, urothelial carcinoma of the bladder; DDR, DNA damage repair; FA, Fanconi Anemia; HR, Homologous Recombination; MMR, Mismatch Repair; NER, Nucleotide Excision Repair.

In our cohort, a total of 122 (49.00%) UC patients, including 66 (50.38%) UTUC and 56 (47.46%) UCB cases, were found to harbor at least one GA in 34 DDR-related genes (**Figure 6A**). Of note, the top prevalently altered genes (**Figure 6B**), respectively in six canonical pathways of Checkpoint, FA, HR, MMR, NER, and others, were *ATM* (N = 24, 9.64%), *BRCA2* (N = 20, 8.03%), *BRCA1* (N = 16, 6.43%), *MSH6* (N = 10, 4.02%), *ERCC2* (N = 16, 6.43%), and *RECQL4* (N = 16, 6.43%). Statistical analysis further disclosed that the frequency of *RECQL4* (9.16% vs. 3.39%), was significantly higher in UTUC; whereas, there were significantly more *ERCC2* (11.02% vs. 2.29%) and *PMS1* (3.39% vs. 0.00%) GAs in UCB (**Figures 6A, B**). GAs distributed in the pathways of HR & others (19.85%) and FA (19.49%) were the most prevalent in UTUC and UCB, respectively (**Figure 6C**). Besides, no significant difference was observed in the distribution of GAs among these six DDR related pathways between UTUC and UCB, with one exception in the pathway of others which was more prevalent in UTUC (19.85% vs. 7.63%, p < 0.01). Additionally, a total of 70 (28.11%) UC patients, including 30 (22.90%) UTUC and 40 (33.90%) UCB patients, had at least one deleterious/likely deleterious GA in DDR related pathways (**Figure 6D**). Whereas, statistical analysis showed that there was no significant difference in the number of deleterious/likely deleterious GAs among these six canonical pathways between UTUC and UCB.

### 3.8 Mutational spectrums and signatures in Chinese UTUC & UCB

**Figure 7:**
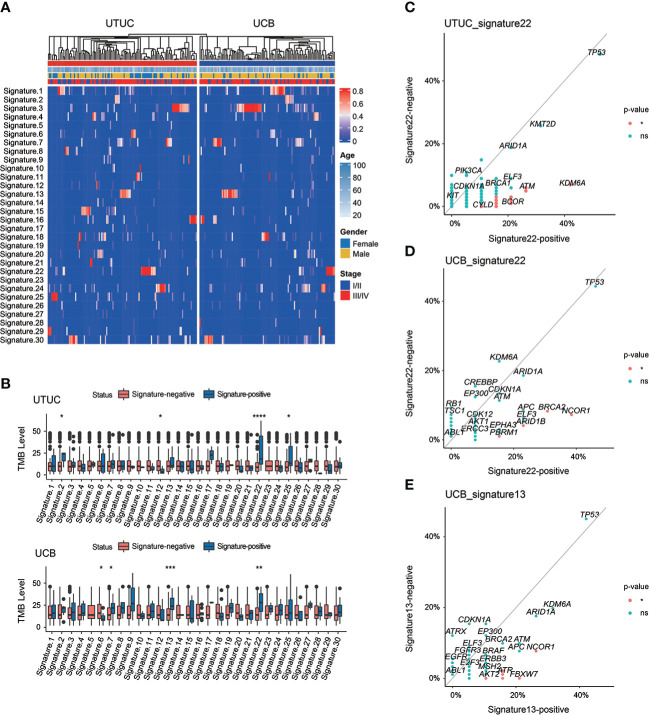
Spectrum of mutational signatures in Chinese UTUC and UCB. **(A)** Comparison of mutational signatures between Chinese UTUC and UCB. **(B)** Comparison of the TMB levels between patients with or without one extent mutational signature, respectively in UTUC and UCB. **(C)** Enrichment of GAs for UTUC patients with or without mutational signature 22. **(D)** Enrichment of GAs for UCB patients with or without mutational signature 22. **(E)** Enrichment of GAs for UCB patients with or without mutational signature 13. *: p < 0.05; **: p < 0.01; ****: p < 0.001; ***: p < 0.0001. ns, not significant.

Known to have a high level of heterogeneity, UTUC and UCB have distinct mutational signatures and etiologies. Of note, it was revealed in our cohort that sig 3 (correlated with failure of DNA double-strand break-repair by HR) was the most prevalent both in UTUC (18.26%) and UCB (34.55%) (**Figure 7A**). Other dominant mutational signatures in UTUC included sig 1 (correlated with spontaneous deamination of 5-methylcytosine), sig 2 (correlated with APOBEC cytidine deaminase, C>T), sig 4 (correlated with exposure to tobacco mutagens), sig 10 (defective DNA mismatch repair), sig 16 (etiology: unknown), and sig 22 (correlated with exposure to AA). On the other hand, sig 1, 4, 13 (correlated with APOBEC-mediated mutagenesis), and 22 were predominantly included in UCB instead. Notably, sig 5 (etiology: unknown) and sig 23 (etiology: unknown) were exclusive to UTUC and UCB samples, respectively, albeit with a low prevalence.

Of note, Chinese UC patients with sig 22, particularly UTUC patients, had markedly elevated TMB level (**Figure 7B**). Besides, significantly differed TMB levels were also identified in Chinese UTUC patients presenting with sig 2, 12 and 25 or UCB patients with sig 6, 7 and 13, respectively (**Figure 7B**). Impressively, the analysis of IMvigor210 cohort further revealed that TMB-high group had the significantly better clinical outcomes, and importantly, TMB-high was markedly correlated with complete/partial responses of immunotherapy (**Supplementary Figure 6**). Moreover, *KDM6A*, *ATM*, *POLD1*, and *BCOR* were more frequently altered in UTUC patients with sig 22 than in those without it (**Figure 7C**). Whereas in UCB, *NCOR1*, *BRCA2*, *ARID1B*, and *PBRM1* were more frequently altered in patients with sig 22 than the counterparts without it (**Figure 7D**). In addition, *NCOR1*, *FBXW7*, *ATR*, *RNF43*, and *AKT2* were more prevalent in UCB patients with sig 13 (**Figure 7E**).

### 3.9 Genomic comparison between chinese and western cohorts

**Figure 8:**
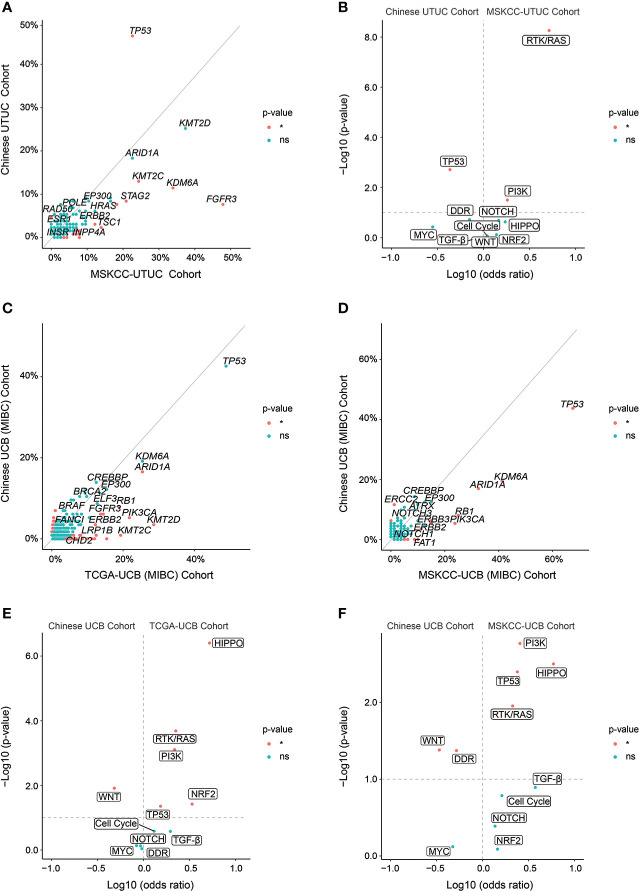
Genomic differences of UTUC or UCB between Chinese and Western cohorts. **(A)** Enrichment of GAs, respectively in Chinese UTUC and MSKCC-UTUC cohorts. **(B)** The frequency of altered molecular pathways [Log_10_ (odds ratio)] by significance [−Log_10_ (p-value)], respectively in Chinese UTUC and MSKCC-UTUC cohorts. Enrichment of GAs, respectively in Chinese UCB and TCGA-UCB cohorts **(C)**, and respectively in Chinese UCB and MSKCC-UCB cohorts **(D)**. The frequency of altered molecular pathways [Log_10_ (odds ratio)] by significance [−Log_10_ (p-value)], respectively in Chinese UCB and TCGA-UCB cohorts **(E)**, and respectively in Chinese UCB and MSKCC-UCB cohorts **(F)**. *: p < 0.05. ns, not significant.

Finally, GA comparison analysis was conducted to reveal the differentiation of molecular characterization of UTUC and UCB between Chinese and Western cohorts. *TP53* was significantly more prevalent in the Chinese UTUC cohort compared to the MSKCC-UTCC cohort; on the contrary *FGFR3*, *KDM6A*, and *KMT2C* were more frequently altered in the MSKCC-UTUC cohort (**Figure 8A**, **Supplementary Table 7**). Accordingly, TP53 signaling pathway was significantly more common in our UTUC cohort; whereas, RTK/RAS and PI3K signaling pathways were more prevalent in the MSKCC-UTUC cohort (**Figure 8B**). In addition, *PPM1D* and *ERCC5* GAs were prevalent in Chinese UCB cohort, compared with TCGA-UCB cohort (**Figure 8C**, **Supplementary Table 8**), but *ERCC2* GAs were frequently altered in our cohort, compared with MSKCC-UCB cohort (**Figure 8D**, **Supplementary Table 9**). In contrast, *ARID1A*, *PIK3CA*, *RB1*, *ERBB2*, and *FAT1* GAs were significantly more prevalent both in the TCGA- and MSKCC-UCB cohort. Pathway enrichment further revealed that WNT signaling pathway was more prevalent in the Chinese UCB cohort when compared to the TCGA-UCB or MSKCC-UCB cohort; whereas, GAs in TP53, RTK/RAS, HIPPO and PI3K signaling pathways were more prevalent in these two Western cohorts (**Figures 8E, F**).

## 4 Discussions

Compared to other kinds of malignancies, UC has the third highest mutational load and a high degree of heterogeneity of GAs following lung carcinoma and melanoma (24). The in-depth interpretation of genomic characteristics discloses biomarkers or signatures, highly unique to certain malignancies, implicating the decision-making of potential targeted therapy and immunotherapy in UC (25). Unfortunately, the majority of Chinese UC patients are usually diagnosed at advanced stages, and there is a dearth of precision treatment strategies. Especially for UTUC patients who are prone to chronic kidney diseases but treated by following the strategies of UCB, as aforementioned, the standard platinum chemotherapy is usually intolerant (15). Hence, understanding the genomic characteristics of Chinese UTUC patients is crucial for therapy development and improving patient outcomes. To the best of our knowledge, this study is composed of the largest Chinese UC sample size, especially UTUC, comprehensively revealing the genomic characterization of UTUC and UCB. Additionally, it is also the first time comparing the GA profiling of renal pelvis with ureter in a Chinese cohort. Overall, the present study might contribute to the development of molecular oncology and provide more opportunities of treatment intervention for UC patients.

Distinct from the Western cohort, GAs in *TP53* gene and TP53-related signaling pathway were significantly more prevalent in the UTUC samples from Chinese cohort, and particularly, patients with ureter tumors harbored more GAs in *TP53* and TP53-related signaling pathway. The tumor-suppressing gene *TP53* plays a central role in the UC development and progression, contributing to genomic instability, anomalous regulation of Cell Cycle and/or apoptotic signaling pathways, and copy number alterations (26). Simultaneously, its alterations are markedly correlated with the high risk of recurrence (27). Altogether, Chinese UTUC patients, especially for those with ureter tumors, may have relatively more invasive tumors and increased risk of disease recurrence than the Western counterparts. The differences in somatic and actionable alterations between the Chinese and Western cohorts together revealed that the mechanisms of mutation-driven carcinogenesis were distinct, from another perspective, genomic characterization would greatly help clinical decision-making of potential targeted therapy and immunotherapy. Identification of clinically significant P/LP germline variants in our cohort also demonstrated that DDR gene alterations were especially frequent, which is consistent with the finding in Western UC patients (28). Nowadays, only LS is regarded as the hereditary cancer syndrome highly correlated with elevated risk of UC, which is caused by germline inactivating alterations in MMR-associated genes (29, 30). It had been found that patients with LS had the increased risk of UTUC up to 12% that was greater than accumulative risk for UCB, particularly for carriers of *MSH2* mutations (31). The incidence of LS in UTUC is rare, while we found in the present study that 2.01%, 3.05% and 0.85% of Chinese UC, UTUC and UCB patients were diagnosed as LS, respectively. This was supported by another Chinese cohort study which revealed that 1.4% UTUC patients were confirmed with LS (32). In two independent western UC cohorts, as reported, 8.77% (28) and over 13.00% (33) of UTUC patients harbored LS-associated gene variants, respectively. Comparatively, Chinese UTUC patients harbored remarkably less germline variants in LS-associated genes. Other than those with LS, 7.63% of Chinese UC patients had other P/LP germline DDR alterations, which were also highly correlated with the carcinogenesis of UC. Furthermore, Germline variants of MMR and other DDR genes might be of guiding significance for treatment selection, as MMR deficiency was correlated with immunotherapy response of PD-L1/PD-1 blockade and *ATM*, *ERCC*2, or other DDR gene alterations were associated with chemotherapy response (34–36). Altogether, it was suggested that germline DDR alterations should be investigated together with somatic alterations profiling, as of therapy response evaluation. In addition, P/LP germline alterations in *ASXL1*, *BAX*, *CDKN2A*, *SDHA*, and *VHL* were rarely reported in Chinese UC patients which was firstly identified in the present study, although P/LP germline alterations in *ASXL1*, *CDKN2A*, *SDHA*, and *VHL* have been reported in the Western UC cohort (28). The molecular characterizations of P/LP germline variants provided the preliminary evidence for the subsequent researches of hereditary UC, and would be of guiding significance to helping establish prevention and surveillance strategies to suppress the incidence of UC.

Furthermore, a total of 122 (49.00%) UC patients harbored alterations in the DDR related signaling pathway, similar to the finding of another Chinese UC cohort (37). Of note, *RECQL4* alterations were significantly more prevalent in Chinese UTUC; whereas, *ERCC2* and *PMS1* alterations were common in UCB. *RECQL4*, as a DNA helicase belonging to the RecQ helicase family, was important in preserving genome stability, thus, its alterations were highly associated with the carcinogenesis (38). As described before, *ERCC2*, known to be involved in the NER pathway for DDR-related mechanism, was markedly associated with chemotherapy or immunotherapy response in UCB. *PSM1*, a component of MMR system, was responsible for the repair of DNA mismatches. The prevalence of *PSM1* was also correlated with therapy response, and its germline variants were usually associated with LS predisposition (39). Moreover, nearly one third of Chinese UC patients, including 33.90% and 22.90% of UCB and UTUC patients, respectively, harbored at least one deleterious/likely deleterious alteration in DDR related pathways. The potential treatment by poly (ADP ribose) polymerase inhibitors (PARPi) could be used for these UC patients with deleterious/likely deleterious DDR related pathway alterations, especially for patients with altered *BRCA1*/*2* presenting with higher sensitivity, and this monotherapy by PARPi inhibitors has approved in clinic for *BRCA1*/*2*-altered breast and ovarian tumors (40). For metastatic UC patients harboring gene mutations in HR, PARPi inhibition also play a potential role to improve clinical outcomes of those receiving Durvalumab plus Olaparib (41). In spite of limited efficacy of monotherapy of PARPi inhibitors, immunotherapy combined with PARPi inhibitors has shown collaborative effects for the treatment of UC patients with alterations in the HR pathway (42). In addition, a pan-cancer study highlighted that altered *PARP1* was correlated with the high degree of immune cell infiltrations, such as CD8+ T cells, in most cancers (43), simultaneously, there were higher expression levels of *PD-1*, *LAG3*, and *CTLA-4* in the *PARP1*-altered group (44, 45).

When comparing the whole genomic profiling of Chinese UTUC with UCB, more alterations in chromatin remodeling related genes (*KMT2A*, *KMT2D*, *KMT2C*), prevalent in Chinese UTUC patients, consistent with the findings in Western cohorts (8, 26). Epigenetic abnormality, such as alterations in chromatin remodeling, DNA methylation, and histone modification, were strongly associated with tumor development and progression (46), meanwhile, epigenetic regulation of chromatin function controlled gene expression pattern (47). Notably, our finding was in consistent with previous studies (37, 48) that the alteration frequency of *FGFR3* was significantly higher in western UTUC patients, which is the main actionable gene in UC. Therefore, Erdafitinib and pemigatinib as effective FGFR inhibitors approved by the FDA-US (49) might be clinically applicable for less patients in China. Notably, *FGFR3* alterations were frequently correlated with the non-T cell inflamed tumor microenvironment which was associated with resistance to immunotherapy (50), besides, frequently altered *FGFR3* was found to be enriched in the luminal UC subtype poorly responding to immunotherapy (51). On the other hand, this might infer that relatively more Chinese UC patients could make responses to the immunotherapy compared with the Western UC patients, due to the lower prevalence of *FGFR3*. However, in light of recently emerging contradictory results, more studies are required to confirm the relationship between FGFR3 and sensitivity to ICIs (52). Besides, *FGFR3* alterations were also correlated with lower responses to platinum-based chemotherapy (53). However, *FGFR3* alterations were closely associated with lower pT stage, tumor grade, and other favorable clinical features and outcomes (54), altogether, it was highly recommended that UC patients with *FGFR3*-altered tumors would be more likely to benefit from anti-FGFR3 therapy. Whereas, platinum-based chemotherapy, immunotherapy, or neoadjuvant chemotherapy was more suitable for UC patients with *FGFR3* wild-type tumors. Moreover, *ERCC2* and *RB1* alterations were prevalent in Chinese UCB, especially for early-stage tumors harboring significantly more alterations in *ERCC2* and *RB1*, of which *ERCC2* might improve the immunotherapy response in UCB (55). Additionally, the inhibition of PPM1D could be a promising anti-tumor strategy to treat *PPM1D*-altered UCB patients (56), and intriguingly, all *PPM1D* alterations were only presented in advanced UCB tumors. Of note, GA analysis of early-stage UTUC and UCB tumors showed that Cell Cycle signaling pathway alterations were quite prevalent in early-stage UCB, probably leading to the increased mutation load which could stimulate tumor progression; on the contrary, accumulative tumor mutations were highly associated with chemotherapy and immunotherapy response. Besides, NOTCH signaling pathway alterations were significantly prevalent in advanced UCB, as known, NOTCH signaling pathway majorly functioned to control cell differentiation and stem cell maintenance (57), of which dysregulation was highly correlated with the epithelial-mesenchymal transition (EMT) in bladder cancer. Therefore, therapies targeting to NOTCH signaling pathway might prevent the EMT of UCB tumors effectively.

Ultimately, this study further revealed the distinct patterns of mutational spectrums in Chinese UTUC & UCB. As acknowledged, characteristic mutational signature within cancer genomes represented specific mutational processes underlying carcinogenesis (58). In the present study, it was found that sig 3, correlated with failure of DNA double-strand break-repair by HR, was the most prevalent both in UTUC (18.26%) and UCB (34.55%) cohorts. However, another Chinese UC cohort demonstrated that sig 1 (41.86%) was predominant in the UTUC cohort (N = 45) and sig 13 (42.99%) was the most frequent in the UCB cohort (N =73) (48), which were also predominant in our cohort. The COSMIC database depicted that sig 1 was an endogenous mutation process attributed to spontaneous deamination of 5-methylcytosine, besides, the AID/APOBEC family of cytidine deaminases converting cytosine to uracil resulted in sig 13. Of note, sig 4 and 22, correlated with exposure to tobacco mutagens and AA, respectively, were observed both in UTUC and UCB cohorts. As known, tobacco exposure is the major risk factor for UC, regardless of UTUC or UCB tumors (59). In addition, a multicenter study had discovered the presence of sig 22 in Chinese UCB, although it only occupied 3% of samples (60), however, the present study revealed that sig 22 was also prevalent among Chinese UCB patients, probably caused by the exposure to AA in traditional Chinese herbal medicine. Altogether, our findings first disclosed that sig 22, correlated with AA exposure, was also highly prevalent in Chinese UCB. Remarkably, it was first time finding that sig 5 (etiology: unknown) and sig 23 (etiology: unknown) were exclusive to a small part of UTUC and UCB samples, respectively, which needed further verifications. Of note, we further disclosed that *KDM6A*, *ATM*, *POLD1*, and *BCOR* alterations were positively associated with sign 22 in UTUC, as demonstrated in the present study, the presence of sig 22 was remarkably correlated with increased TMB level. Indeed, both of *KDM6A* (61) and *ATM* (62) alterations were markedly associated with a higher level of TMB, whereas, it was firstly identified that *POLD1* and *BCOR* alterations were also correlated with increased TMB level as well as sig 22 in UTUC. Besides that *BRCA2* (63) and *PBRM1* (64) alterations might lead to the elevated TMB level, also it was first time revealing that *NCOR1* and *ARID1B* alterations were associated with TMB level and sig 22 in UCB. Regarding patients with sig 13, *NCOR1*, *FBXW7*, *ATR*, *RNF43*, and *AKT2* alterations were abundantly enriched in our UCB cohort, as acknowledged, all of which were first found to be correlated with occurring sig 13 and increased TMB level in UCB. TMB level was positively correlated with immunotherapy response in UC (65), moreover, the IMvigor210 cohort directly revealed that TMB level was positively correlated with not only immunotherapy response but also clinical outcomes. In summary, these identified altered genes in the present study had potential roles as biomarkers for immunotherapy response and we further confirmed that Chinese UTUC and UCB patients had distinct patterns of mutational spectrums, and mutational characteristics.

Even though, to the best of our knowledge, this study has analyzed the genomic landscape of the greatest number of Chinese UTUC cases to date, the limited sample size and retrospective nature of study design may have influenced our findings. Moreover, there were differences between our sequencing methods and those with which we compared even under the same detected gene spectrum, which may potentially have affected the results. Therefore, further research with expanded sample size may be necessary to validate the findings.

## 5 Conclusions

In the present study, the systematic investigation of germline variants first proposed that germline alterations in Chinese UC occur predominantly in DDR-related genes. Moreover, comprehensive genomic characterizations of Chinese UTUC and UCB provided more and deeper insights of distinct pathogenetic mechanisms, and offered more precision therapeutic regimens for different UC patients.

## Data availability statement

The data presented in the study are deposited in the National Genomics Data Center, China National Center for Bioinformation / Beijing Institute of Genomics, Chinese Academy of Sciences under the GSA-human accession number: HRA003808.

## Ethics statement

Written informed consents of all involved patients have been collected, and this study was conducted according to the International Ethical Guidelines for Biomedical Research Involving Human Subjects, the Declaration of Helsinki, and approved by the ethics committee of Peking University First Hospital.

## Author contributions

QT, LZ, and XL proposed this study. QT & WZ collected samples. CW, SX, CX, CY, and QS collected raw data and made bioinformatic analyses. QT, WZ, and CW produced figures and tables. QT & WZ drafted original manuscript. QT, LZ, and XL revised manuscript. All authors contributed to the article and approved the submitted version.

## References

[1] Antoni S Ferlay J Soerjomataram I Znaor A Jemal A Bray F. Bladder cancer incidence and mortality: A global overview and recent trends. Eur Urol (2017) 71(1):96–108. doi: 10.1016/j.eururo.2016.06.010 27370177

[2] Sung H Ferlay J Siegel RL Laversanne M Soerjomataram I Jemal A. Global cancer statistics 2020: GLOBOCAN estimates of incidence and mortality worldwide for 36 cancers in 185 countries. CA: A Cancer J Clin (2021) 71(3):209–49. doi: 10.3322/caac.21660 33538338

[3] Zheng R Zhang S Zeng H Wang S Sun K Chen R. Cancer incidence and mortality in China, 2016. J Natl Cancer Center (2022). 2 (1): 1-9 doi: 10.1016/j.jncc.2022.02.002 PMC11256658 39035212

[4] Green D Rink M Xylinas E Matin S Stenzl A Roupret M. Urothelial carcinoma of the bladder and the upper tract: disparate twins. J Urol (2013) 189(4):1214–21. doi: 10.1016/j.juro.2012.05.079 23023150

[5] Necchi A Pal SK Ross JS Madison R Agarwal N Sonpavde G. Comprehensive genomic profiling (CGP) of upper-tract (UTUC) and bladder (BUC) urothelial carcinoma reveals opportunities for therapeutic and biomarker development. J Clin Oncol (2019) 37(15_suppl):4581–1. doi: 10.1200/JCO.2019.37.15_suppl.4581

[6] Glaser AP Fantini D Shilatifard A Schaeffer EM Meeks JJ. The evolving genomic landscape of urothelial carcinoma. Nat Rev Urol (2017) 14(4):215–29. doi: 10.1038/nrurol.2017.11 28169993

[7] Weinstein JN Akbani R Broom BM Wang W Verhaak RGW McConkey D. Comprehensive molecular characterization of urothelial bladder carcinoma. Nature (2014) 507(7492):315–22. doi: 10.1038/nature12965 PMC3962515 24476821

[8] Audenet F Isharwal S Cha EK Donoghue MTA Drill EN Ostrovnaya I. Clonal relatedness and mutational differences between upper tract and bladder urothelial carcinoma. Clin Cancer Res (2019) 25(3):967–76. doi: 10.1158/1078-0432.CCR-18-2039 PMC6359971 30352907

[9] Necchi A Madison R Pal SK Ross JS Agarwal N Sonpavde G. Comprehensive genomic profiling of upper-tract and bladder urothelial carcinoma. Eur Urol Focus (2021) 7(6):1339–46. doi: 10.1016/j.euf.2020.08.001 32861617

[10] Nassar AH Umeton R Kim J Lundgren K Harshman L Van Allen EM. Mutational analysis of 472 urothelial carcinoma across grades and anatomic sites. Clin Cancer Res (2019) 25(8):2458–70. doi: 10.1158/1078-0432.CCR-18-3147 30593515

[11] Teo MY Bambury RM Zabor EC Jordan E Al-Ahmadie H Boyd ME. DNA Damage response and repair gene alterations are associated with improved survival in patients with platinum-treated advanced urothelial carcinoma. Clin Cancer Res (2017) 23(14):3610–8. doi: 10.1158/1078-0432.CCR-16-2520 PMC5511570 28137924

[12] Teo MY Seier K Ostrovnaya I Regazzi AM Kania BE Moran MM. Alterations in DNA damage response and repair genes as potential marker of clinical benefit from PD-1/PD-L1 blockade in advanced urothelial cancers. J Clin Oncol (2018) 36(17):1685–94. doi: 10.1200/JCO.2017.75.7740 PMC6366295 29489427

[13] Colin P Koenig P Ouzzane A Berthon N Villers A Biserte J. Environmental factors involved in carcinogenesis of urothelial cell carcinomas of the upper urinary tract. BJU Int (2009) 104(10):1436–40. doi: 10.1111/j.1464-410X.2009.08838.x 19689473

[14] Grollman AP Shibutani S Moriya M Miller F Wu L Moll U. Aristolochic acid and the etiology of endemic (Balkan) nephropathy. Proc Natl Acad Sci (2007) 104(29):12129–34. doi: 10.1073/pnas.0701248104 PMC1913550 17620607

[15] Xiong G Chen X Li X Fang D Zhang L Yang L. Prevalence and factors associated with baseline chronic kidney disease in China: A 10-year study of 785 upper urinary tract urothelial carcinoma patients. J Formosan Med Assoc (2014) 113(8):521–6. doi: 10.1016/j.jfma.2013.04.001 23684217

[16] Crockett DG Wagner DG Holmäng S Johansson SL Lynch HT. Upper urinary tract carcinoma in lynch syndrome cases. J Urol (2011) 185(5):1627–30. doi: 10.1016/j.juro.2010.12.102 21419447

[17] Kloor M Voigt AY Schackert HK Schirmacher P Doeberitz M Bläker H. Analysis of EPCAM protein expression in diagnostics of lynch syndrome. J Clin Oncol (2011) 29(2):223–7. doi: 10.1200/JCO.2010.32.0820 21115857

[18] Sanchez-Vega F Mina M Armenia J Chatila WK Luna A La KC. Oncogenic signaling pathways in the cancer genome atlas. Cell (2018) 173(2):321–337.e310. doi: 10.1016/j.cell.2018.03.035 29625050 PMC6070353

[19] Alexandrov LB Nik-Zainal S Wedge DC Aparicio SAJR Behjati S Biankin AV. Signatures of mutational processes in human cancer. Nature (2013) 500(7463):415–21. doi: 10.1038/nature12477 PMC3776390 23945592

[20] Sfakianos JP Kim PH Iyer G Cha EK Zabor EC Hakimi AA. Targeted sequencing of upper tract urothelial carcinoma. J Clin Oncol (2014) 32(4_suppl):309–9. doi: 10.1200/jco.2014.32.4_suppl.309

[21] Liu J Lichtenberg T Hoadley KA Poisson LM Lazar AJ Cherniack AD. An integrated TCGA pan-cancer clinical data resource to drive high-quality survival outcome analytics. Cell (2018) 173(2):400–416.e411. doi: 10.1016/j.cell.2018.02.052 29625055 PMC6066282

[22] Kim PH Cha EK Sfakianos JP Iyer G Zabor EC Scott SN. Genomic predictors of survival in patients with high-grade urothelial carcinoma of the bladder. Eur Urol (2015) 67(2):198–201. doi: 10.1016/j.eururo.2014.06.050 25092538 PMC4312739

[23] Balar AV Galsky MD Rosenberg JE Powles T Petrylak DP Bellmunt J. Atezolizumab as first-line treatment in cisplatin-ineligible patients with locally advanced and metastatic urothelial carcinoma: a single-arm, multicentre, phase 2 trial. Lancet (2017) 389(10064):67–76. doi: 10.1016/S0140-6736(16)32455-2 27939400 PMC5568632

[24] Lawrence MS Stojanov P Polak P Kryukov GV Cibulskis K Sivachenko A. Mutational heterogeneity in cancer and the search for new cancer-associated genes. Nature (2013) 499(7457):214–8. doi: 10.1038/nature12213 PMC3919509 23770567

[25] Roberts SA Gordenin DA. Hypermutation in human cancer genomes: footprints and mechanisms. Nat Rev Cancer (2014) 14(12):786–800. doi: 10.1038/nrc3816 25568919 PMC4280484

[26] Moss TJ Qi Y Xi L Peng B Kim T-B Ezzedine NE. Comprehensive genomic characterization of upper tract urothelial carcinoma. Eur Urol (2017) 72(4):641–9. doi: 10.1016/j.eururo.2017.05.048 28601352

[27] Collin-Chavagnac D Marçais C Billon S Descotes F Piaton E Decaussin M. Quantitative loss of heterozygosity analysis for urothelial carcinoma detection and prognosis. Urology (2010) 76(2):515.e511–515.e517. doi: 10.1016/j.urology.2009.11.046 20206968

[28] Carlo MI Ravichandran V Srinavasan P Bandlamudi C Kemel Y Ceyhan-Birsoy O. Cancer susceptibility mutations in patients with urothelial malignancies. J Clin Oncol (2020) 38(5):406–14. doi: 10.1200/JCO.19.01395 PMC7351337 31794323

[29] Harper HL McKenney JK Heald B Stephenson A Campbell SC Plesec T. Upper tract urothelial carcinomas: frequency of association with mismatch repair protein loss and lynch syndrome. Modern Pathol (2017) 30(1):146–56. doi: 10.1038/modpathol.2016.171 27713421

[30] Latham A Srinivasan P Kemel Y Shia J Bandlamudi C Mandelker D. Microsatellite instability is associated with the presence of lynch syndrome pan-cancer. J Clin Oncol (2019) 37(4):286–95. doi: 10.1200/JCO.18.00283 PMC6553803 30376427

[31] Joost P Therkildsen C Dominguez-Valentin M Jönsson M Nilbert M. Urinary tract cancer in lynch syndrome; increased risk in carriers of MSH2 mutations. Urology (2015) 86(6):1212–7. doi: 10.1016/j.urology.2015.08.018 26385421

[32] Guan B Wang J Li X Lin L Fang D Kong W. Identification of germline mutations in upper tract urothelial carcinoma with suspected lynch syndrome. Front Oncol (2022) 12. doi: 10.3389/fonc.2022.774202 PMC8966221 35372080

[33] Nassar AH Abou Alaiwi S AlDubayan SH Moore N Mouw KW Kwiatkowski DJ. Prevalence of pathogenic germline cancer risk variants in high-risk urothelial carcinoma. Genet Med (2020) 22(4):709–18. doi: 10.1038/s41436-019-0720-x PMC7118025 31844177

[34] Yap K Kiyotani K Tamura K Antic T Jang M Montoya M. Whole-exome sequencing of muscle-invasive bladder cancer identifies recurrent mutations of UNC5C and prognostic importance of DNA repair gene mutations on survival. Clin Cancer Res (2014) 20(24):6605–17. doi: 10.1158/1078-0432.CCR-14-0257 PMC4268280 25316812

[35] Liu D Plimack E Hoffman-Censits J Garraway L Bellmunt J Van Allen E. Clinical validation of chemotherapy response biomarker ERCC2 in muscle-invasive urothelial bladder carcinoma. JAMA Oncol (2016) 2(8):1094–6. doi: 10.1001/jamaoncol.2016.1056 PMC5515075 27310333

[36] Le D Uram J Wang H Bartlett B Kemberling H Eyring A. PD-1 blockade in tumors with mismatch-repair deficiency. New Engl J Med (2015) 372(26):2509–20. doi: 10.1056/NEJMoa1500596 PMC4481136 26028255

[37] Yang B Zhao X Wan C Ma X Niu S Guo A. Genomic profiling of Chinese patients with urothelial carcinoma. BMC Cancer (2021) 21(1):162. doi: 10.1186/s12885-021-07829-1 33588785 PMC7885246

[38] Mo D Zhao Y Balajee AS. Human RecQL4 helicase plays multifaceted roles in the genomic stability of normal and cancer cells. Cancer Lett (2018) 413:1–10. doi: 10.1016/j.canlet.2017.10.021 29080750

[39] Peltomäki P. Lynch syndrome genes. Familial Cancer (2005) 4(3):227–32. doi: 10.1007/s10689-004-7993-0 16136382

[40] Trenner A Sartori AA. Harnessing DNA double-strand break repair for cancer treatment. Front Oncol (2019) 9. doi: 10.3389/fonc.2019.01388 PMC6921965 31921645

[41] Rosenberg JE Park SH Kozlov V Dao TV Castellano D Li J-R. Durvalumab plus olaparib in previously untreated, platinum-ineligible patients with metastatic urothelial carcinoma: A multicenter, randomized, phase II trial (BAYOU). J Clin Oncol (2022) JCO.22.00205. doi: 10.1200/jco.22.00205 PMC9788981 35737919

[42] Powles T Carroll D Chowdhury S Gravis G Joly F Carles J. An adaptive, biomarker-directed platform study of durvalumab in combination with targeted therapies in advanced urothelial cancer. Nat Med (2021) 27(5):793–801. doi: 10.1038/s41591-021-01317-6 33941921

[43] Zhang X Wang Y AG Qu C Chen J. Pan-cancer analysis of PARP1 alterations as biomarkers in the prediction of immunotherapeutic effects and the association of its expression levels and immunotherapy signatures. Front Immunol (2021) 12. doi: 10.3389/fimmu.2021.721030 PMC8438309 34531868

[44] Stewart RA Pilié PG Yap TA. Development of PARP and immune-checkpoint inhibitor combinations. Cancer Res (2018) 78(24):6717–25. doi: 10.1158/0008-5472.CAN-18-2652 30498083

[45] Vidotto T Nersesian S Graham C Siemens DR Koti M. DNA Damage repair gene mutations and their association with tumor immune regulatory gene expression in muscle invasive bladder cancer subtypes. J ImmunoTherapy Cancer (2019) 7(1):148. doi: 10.1186/s40425-019-0619-8 PMC6556053 31174611

[46] Esteller M. Epigenetics in cancer. New Engl J Med (2008) 358(11):1148–59. doi: 10.1056/NEJMra072067 18337604

[47] Frew IJ Timmers HTM Schüle R Gratzke C. The complex genetics of epigenetics in urothelial carcinomas. Nat Rev Urol (2020) 17(12):655–6. doi: 10.1038/s41585-020-00386-5 33037422

[48] Yang K Yu W Liu H Ding F Zhang Y Zhang Y. Comparison of genomic characterization in upper tract urothelial carcinoma and urothelial carcinoma of the bladder. Oncologist (2021) 26(8):e1395–405. doi: 10.1002/onco.13839 PMC8342585 34050578

[49] Roskoski R. The role of fibroblast growth factor receptor (FGFR) protein-tyrosine kinase inhibitors in the treatment of cancers including those of the urinary bladder. Pharmacol Res (2020) 151:104567. doi: 10.1016/j.phrs.2019.104567 31770593

[50] Sweis R Spranger S Bao R Paner G Stadler W Steinberg G. Molecular drivers of the non-T-cell-Inflamed tumor microenvironment in urothelial bladder cancer. Cancer Immunol Res (2016) 4(7):563–8. doi: 10.1158/2326-6066.CIR-15-0274 PMC4943758 27197067

[51] Robertson A Kim J Al-Ahmadie H Bellmunt J Guo G Cherniack A. Comprehensive molecular characterization of muscle-invasive bladder cancer. Cell (2017) 171(3):540–556.e525. doi: 10.1016/j.cell.2017.09.007 28988769 PMC5687509

[52] Wang L Gong Y Saci A Szabo PM Martini A Necchi A. Fibroblast growth factor receptor 3 alterations and response to PD-1/PD-L1 blockade in patients with metastatic urothelial cancer. Eur Urol (2019) 76(5):599–603. doi: 10.1016/j.eururo.2019.06.025 31272788 PMC6801024

[53] Teo MY Mota JM Whiting KA Li HA Funt SA Lee C-H. Fibroblast growth factor receptor 3 alteration status is associated with differential sensitivity to platinum-based chemotherapy in locally advanced and metastatic urothelial carcinoma. Eur Urol (2020) 78(6):907–15. doi: 10.1016/j.eururo.2020.07.018 PMC8215618 32753285

[54] van Rhijn BWG Mertens LS Mayr R Bostrom PJ Real FX Zwarthoff EC. FGFR3 mutation status and FGFR3 expression in a Large bladder cancer cohort treated by radical cystectomy: Implications for anti-FGFR3 treatment?†. Eur Urol (2020) 78(5):682–7. doi: 10.1016/j.eururo.2020.07.002 32682615

[55] Ikeda S Hansel DE Kurzrock R. Beyond conventional chemotherapy: Emerging molecular targeted and immunotherapy strategies in urothelial carcinoma. Cancer Treat Rev (2015) 41(8):699–706. doi: 10.1016/j.ctrv.2015.06.004 26138514

[56] Deng W Li J Dorrah K Jimenez-Tapia D Arriaga B Hao Q. The role of PPM1D in cancer and advances in studies of its inhibitors. Biomedicine Pharmacotherapy (2020) 125:109956. doi: 10.1016/j.biopha.2020.109956 32006900 PMC7080581

[57] Greife A Hoffmann MJ Schulz WA. Consequences of disrupted notch signaling in bladder cancer. Eur Urol (2015) 68(1):3–4. doi: 10.1016/j.eururo.2015.02.034 25791514

[58] Alexandrov LB Kim J Haradhvala NJ Huang MN Tian Ng AW Wu Y. The repertoire of mutational signatures in human cancer. Nature (2020) 578(7793):94–101. doi: 10.1038/s41586-020-1943-3 32025018 PMC7054213

[59] Edwards DC Yankelevich GR Dreher PC Narowska G Kim D Taylor Z. Socio-environmental conditions associated with geospatial clusters of urothelial carcinoma: A multi-institutional analysis. J Clin Oncol (2021) 39(6_suppl):392–2. doi: 10.1200/JCO.2021.39.6_suppl.392 34288718

[60] Poon S Huang M Choo Y McPherson J Yu W Heng H. Mutation signatures implicate aristolochic acid in bladder cancer development. Genome Med (2015) 7(1):38. doi: 10.1186/s13073-015-0161-3 26015808 PMC4443665

[61] Jia Y He N Yang Y Huang Y Zhang X Fu Z. Tumor mutation burden and immune microenvironment analysis of urothelial carcinoma. J Clin Oncol (2021) 39(6_suppl):494–4. doi: 10.1200/JCO.2021.39.6_suppl.494

[62] Joshi M Grivas P Mortazavi A Monk P Clinton SK Woo MS-A. Alterations of DNA damage response (DDR) genes correlate with favorable response and overall survival (OS) in anti-PD-1/PD-L1-treated advanced urothelial cancer (UC). J Clin Oncol (2019) 37(7_suppl):438–8. doi: 10.1200/JCO.2019.37.7_suppl.438

[63] Bellmunt J Kim J Reardon B Perera-Bel J Orsola A Rodriguez-Vida A. Genomic predictors of good outcome, recurrence, or progression in high-grade T1 non–Muscle-Invasive bladder cancer. Cancer Res (2020) 80(20):4476–86. doi: 10.1158/0008-5472.CAN-20-0977 PMC9361191 32868381

[64] Yang Q Shen R Xu H Shi X Xu L Zhang L. Comprehensive analyses of PBRM1 in multiple cancer types and its association with clinical response to immunotherapy and immune infiltrates. Ann Trans Med (2021) 9(6):465. doi: 10.21037/atm-21-289 PMC8039713 33850862

[65] Sheng X Chen H Hu B Yao X Liu Z Yao X. Safety, efficacy, and biomarker analysis of toripalimab in patients with previously treated advanced urothelial carcinoma: Results from a multicenter phase II trial POLARIS-03. Clin Cancer Res (2022) 28(3):489–97. doi: 10.1158/1078-0432.CCR-21-2210 34740921

