# Supplementary material for: Comprehensive genomic profiling of upper tract urothelial carcinoma and urothelial carcinoma of the bladder identifies distinct molecular characterizations with potential implications for targeted therapy & immunotherapy
Source: Front Immunol. 2023 Feb 3;13:1097730. doi: 10.3389/fimmu.2022.1097730 (PMC9936149; doi:10.3389/fimmu.2022.1097730)
Supplement: Supplementary file 1 [file Image_1.pdf]

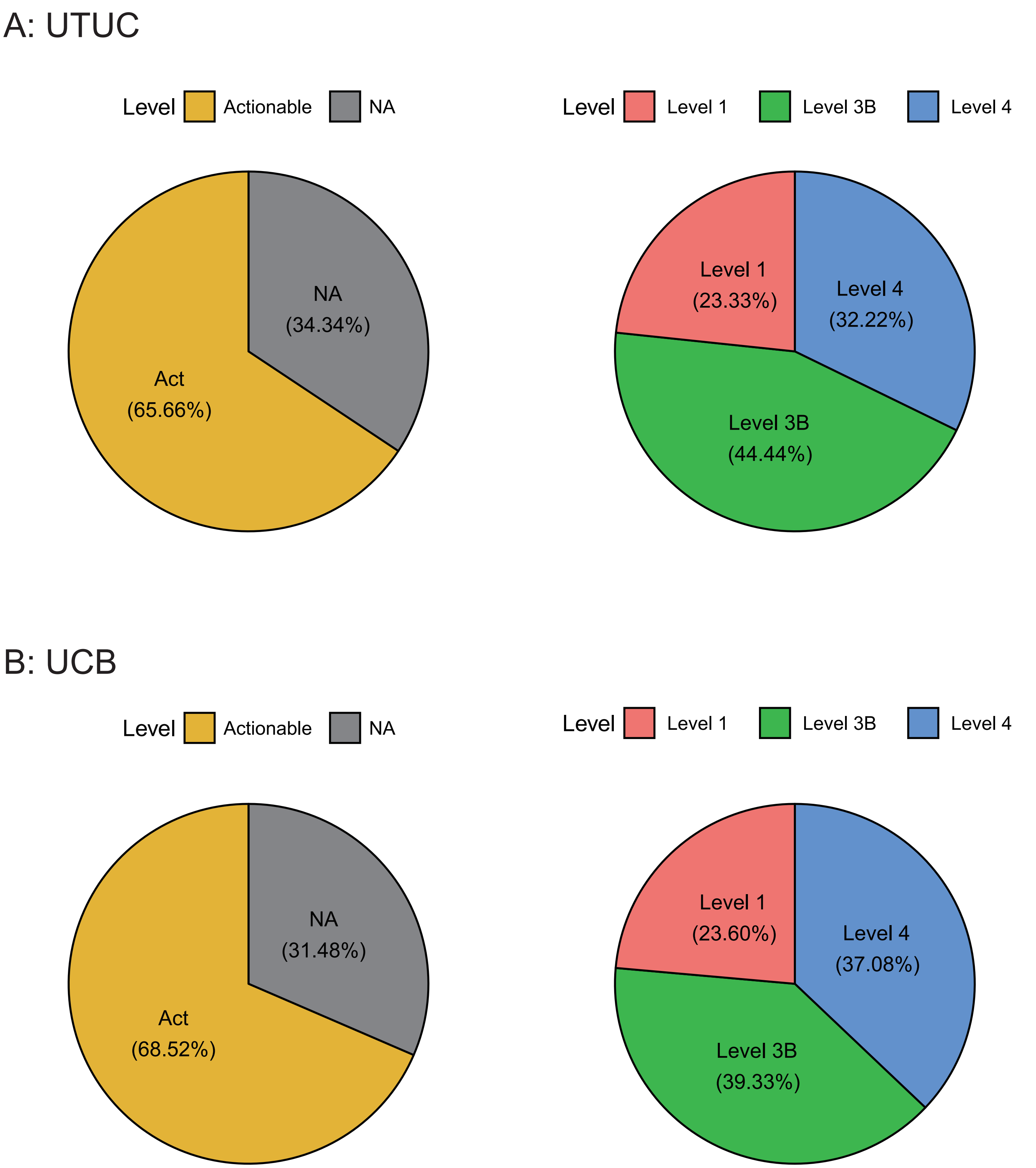

A: UTUC
Level
Actionable
NA
Level
Level 1
Level 3B
Level 4
NA
(34.34%)
Act
(65.66%)
Level 1
(23.33%)
Level 4
(32.22%)
Level 3B
(44.44%)
B: UCB
Level
Actionable
NA
Level
Level 1
Level 3B
Level 4
NA
(31.48%)
Act
(68.52%)
Level 1
(23.60%)
Level 4
(37.08%)
Level 3B
(39.33%)
