# Supplementary material for: Comprehensive genomic profiling of upper tract urothelial carcinoma and urothelial carcinoma of the bladder identifies distinct molecular characterizations with potential implications for targeted therapy & immunotherapy
Source: Front Immunol. 2023 Feb 3;13:1097730. doi: 10.3389/fimmu.2022.1097730 (PMC9936149; doi:10.3389/fimmu.2022.1097730)
Supplement: Supplementary file 2 [file Image_2.pdf]

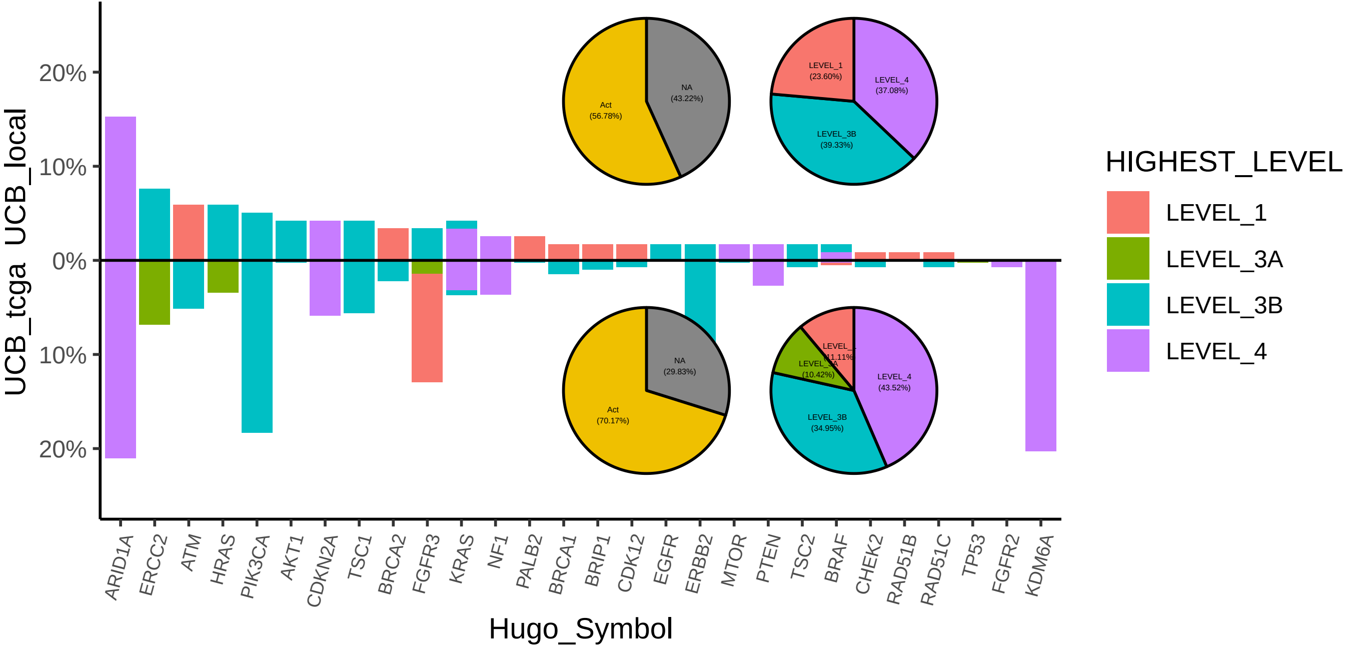

UCB_tcga UCB_local
20%
10%
0%
10%
20%
Hugo_Symbol
ARID1A
ERCC2
ATM
HRAS
PIK3CA
AKT1
CDKN2A
TSC1
BRCA2
FGFR3
KRAS
NF1
PALB2
BRCA1
BRIP1
CDK12
EGFR
ERBB2
MTOR
PTEN
TSC2
BRAF
CHEK2
RAD51B
RAD51C
TP53
FGFR2
KDM6A
Act (56.78%)
NA (43.22%)
LEVEL_1 (23.60%)
LEVEL_4 (37.08%)
LEVEL_3B (39.33%)
Act (70.17%)
NA (29.83%)
LEVEL_4 (43.52%)
LEVEL_3B (34.95%)
(10.42%)
HIGHEST_LEVEL
LEVEL_1
LEVEL_3A
LEVEL_3B
LEVEL_4
