# Supplementary figures and images for: Comprehensive genomic profiling of upper tract urothelial carcinoma and urothelial carcinoma of the bladder identifies distinct molecular characterizations with potential implications for targeted therapy & immunotherapy
Source: Front Immunol. 2023 Feb 3;13:1097730. doi: 10.3389/fimmu.2022.1097730 (PMC9936149; doi:10.3389/fimmu.2022.1097730)

UCB\_MSK UCB\_local

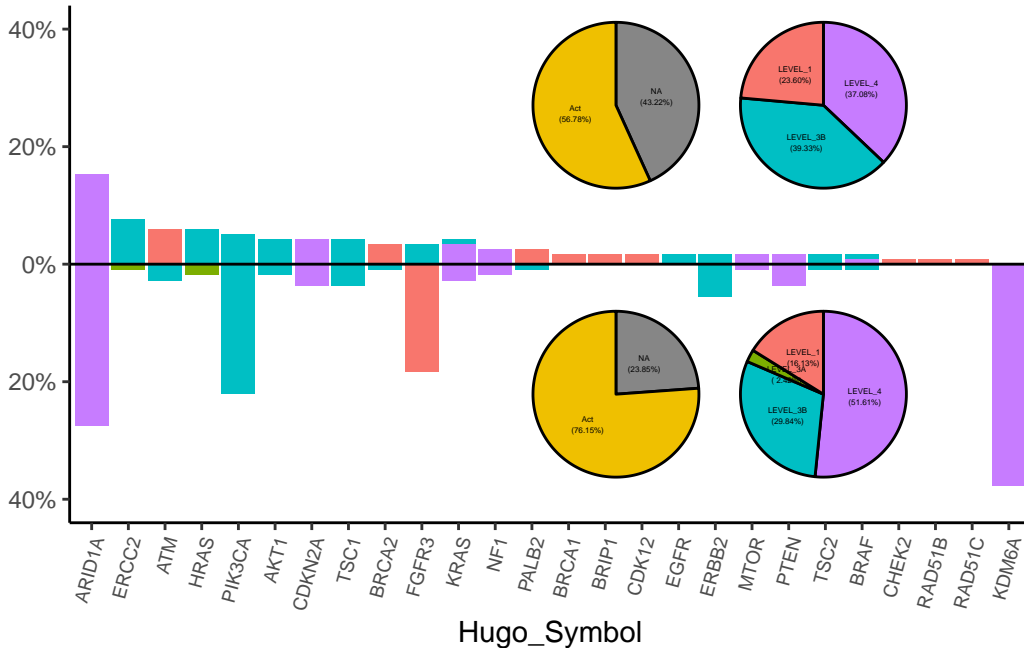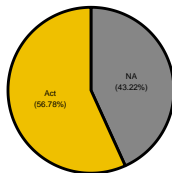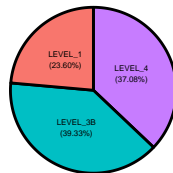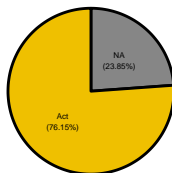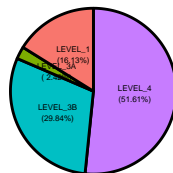

Supplement: Supplementary file 3 [file Image_3.pdf]

UTUC\_msk UTUC\_local

20%  
0%  
20%  
40%

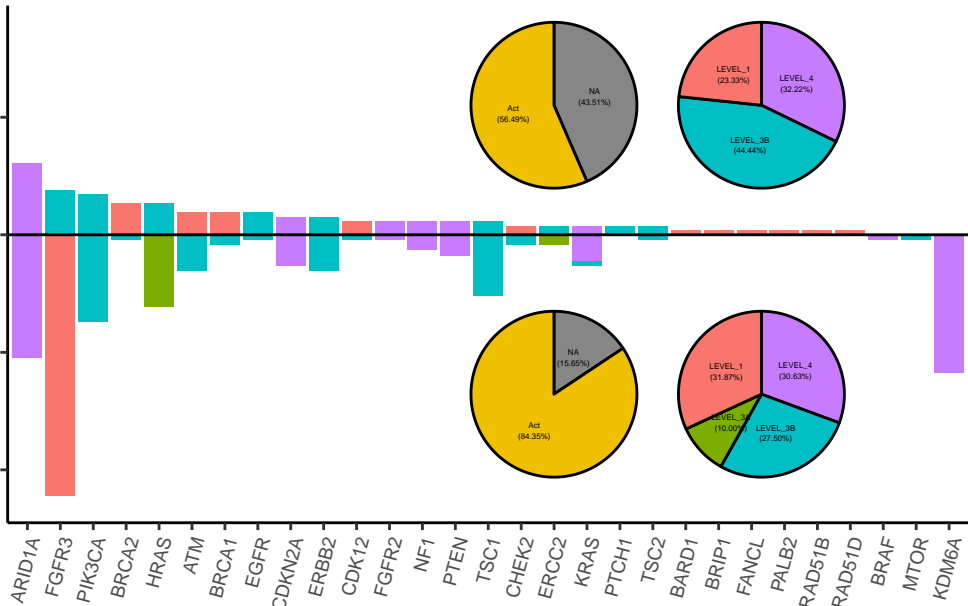

HIGHEST\_LEVEL

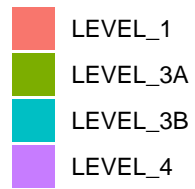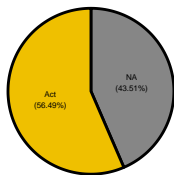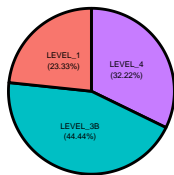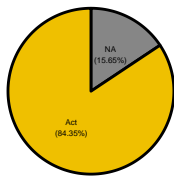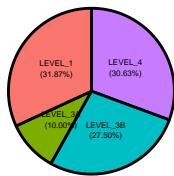

Supplement: Supplementary file 4 [file Image_4.pdf]

A

UTUC:I/II

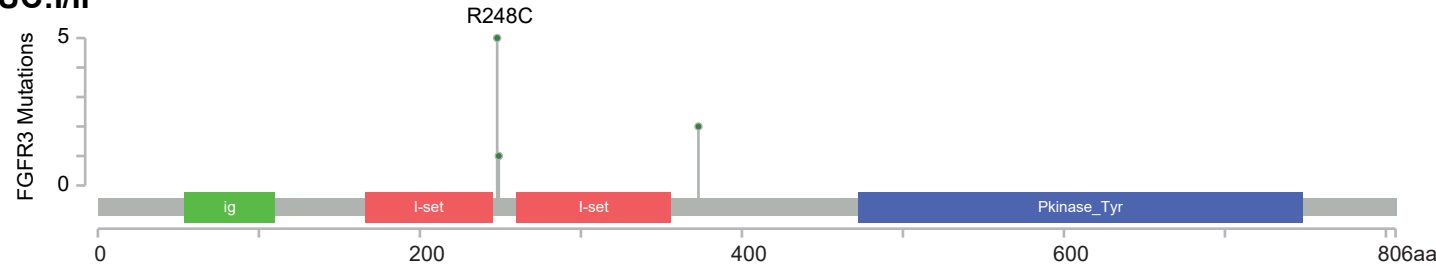

UCB:I/II

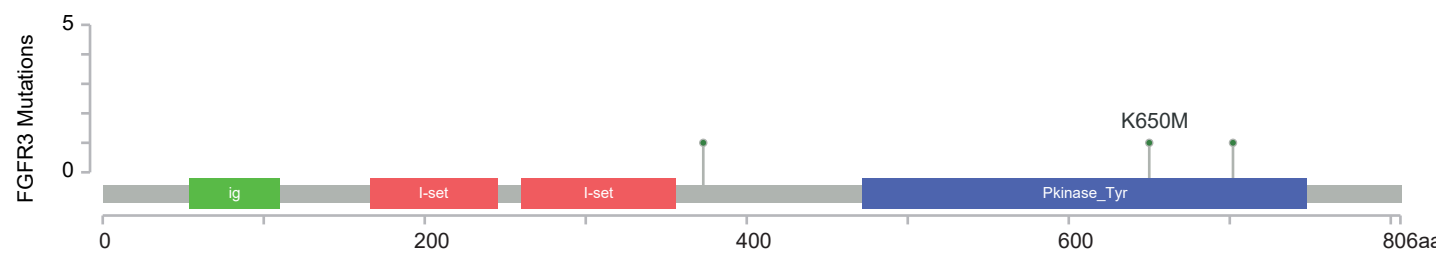

B

UTUC:III/IV

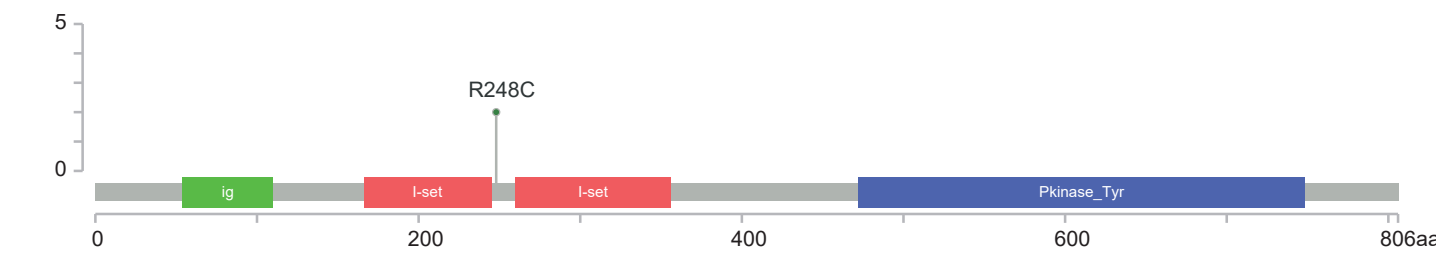

UCB:III/IV

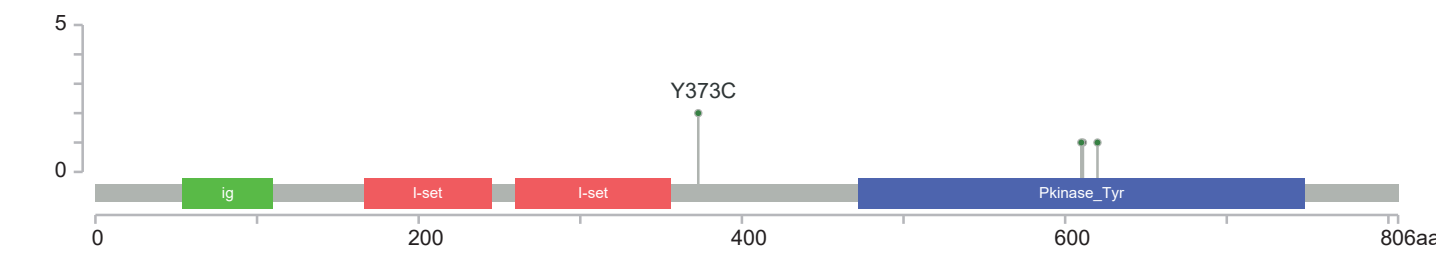

Supplement: Supplementary file 5 [file Image_5.pdf]

binaryResponse CR/PR SD/PD

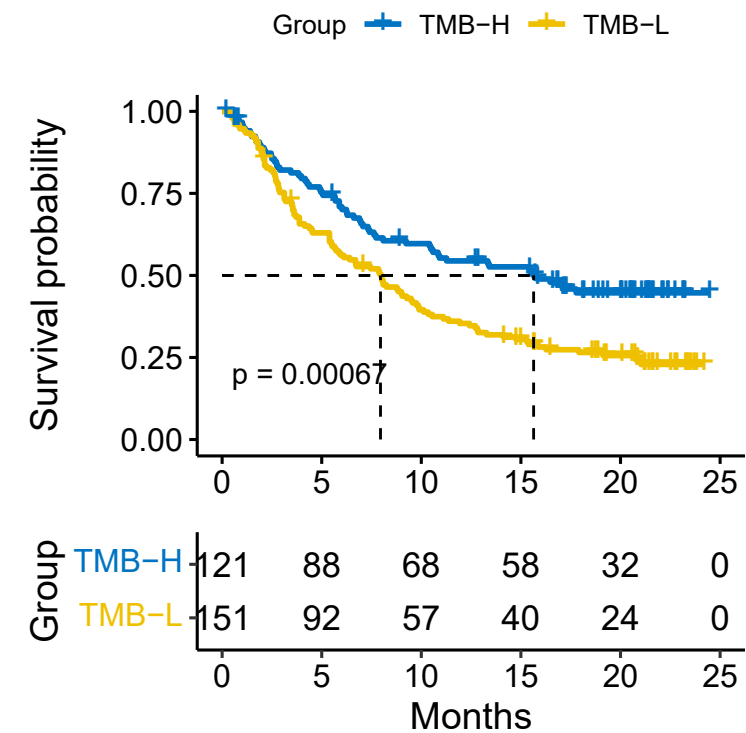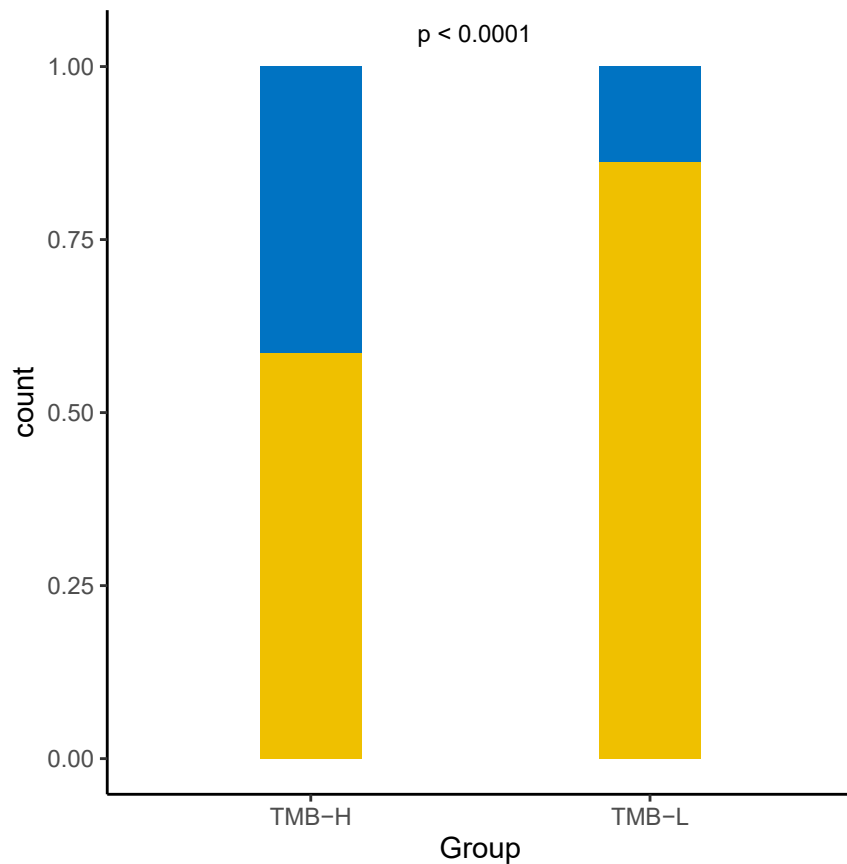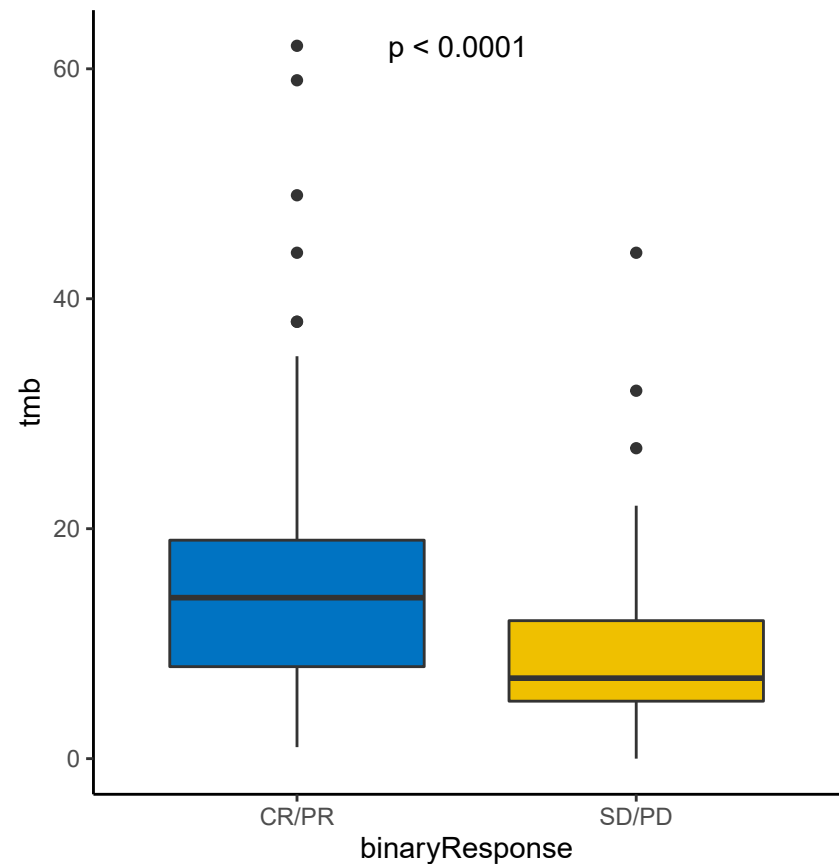

Supplement: Supplementary file 6 [file Image_6.pdf]
