# Supplementary material for: Comprehensive genomic profiling of upper tract urothelial carcinoma and urothelial carcinoma of the bladder identifies distinct molecular characterizations with potential implications for targeted therapy & immunotherapy
Source: Front Immunol. 2023 Feb 3;13:1097730. doi: 10.3389/fimmu.2022.1097730 (PMC9936149; doi:10.3389/fimmu.2022.1097730)
Supplement: Supplementary file 7 [file Table_1.docx]

**Supplementary Table 1.** A panel of 618 genes sequenced in this study

| *ABL1* | *CHEK1* | *FOXA1* | *KMT2B* | *PIK3CB* | *SHH* |
| --- | --- | --- | --- | --- | --- |
| *ABL2* | *CHEK2* | *FOXA2* | *KMT2C* | *PIK3CD* | *SHOC2* |
| *ACVR1* | *CIC* | *FOXL2* | *KMT2D* | *PIK3CG* | *SHQ1* |
| *ACVR1B* | *CREBBP* | *FOXO1* | *KNSTRN* | *PIK3R1* | *SLC16A7* |
| *AGO2* | *CRKL* | *FOXP1* | *KRAS* | *PIK3R2* | *SLC19A1* |
| *AIP* | *CRLF2* | *FRS2* | *LATS1* | *PIK3R3* | *SLIT2* |
| *AKT1* | *CSDE1* | *FUBP1* | *LATS2* | *PIM1* | *SLX4* |
| *AKT2* | *CSF1R* | *FYN* | *LMO1* | *PLCG2* | *SMAD2* |
| *AKT3* | *CSF3R* | *GABRA6* | *LRP1B* | *PLK1* | *SMAD3* |
| *ALK* | *CTCF* | *GATA1* | *LRRK2* | *PLK2* | *SMAD4* |
| *ALOX12B* | *CTLA4* | *GATA2* | *LYN* | *PMAIP1* | *SMARCA1* |
| *AMER1* | *CTNNA1* | *GATA3* | *LZTR1* | *PMS1* | *SMARCA4* |
| *ANKRD11* | *CTNNB1* | *GATA4* | *MAGI2* | *PMS2* | *SMARCB1* |
| *APC* | *CUL3* | *GATA6* | *MALT1* | *PNRC1* | *SMARCD1* |
| *AR* | *CXCR4* | *GID4* | *MAP2K1* | *POLD1* | *SMO* |
| *ARAF* | *CYLD* | *GLI1* | *MAP2K2* | *POLE* | *SMYD3* |
| *ARFRP1* | *CYSLTR2* | *GLI2* | *MAP2K4* | *PPARG* | *SNAI1* |
| *ARID1A* | *DAXX* | *GNA11* | *MAP3K1* | *PPM1D* | *SNAI2* |
| *ARID1B* | *DCUN1D1* | *GNA13* | *MAP3K13* | *PPP2R1A* | *SNCAIP* |
| *ARID2* | *DDB2* | *GNAQ* | *MAP3K14* | *PPP4R2* | *SOCS1* |
| *ARID5B* | *DDR2* | *GNAS* | *MAP4K1* | *PPP6C* | *SOS1* |
| *ASXL1* | *DICER1* | *GOPC* | *MAPK1* | *PRDM1* | *SOX10* |
| *ASXL2* | *DIRAS3* | *GPC3* | *MAPK3* | *PRDM14* | *SOX17* |
| *ATM* | *DIS3* | *GPR124* | *MAPKAP1* | *PREX2* | *SOX2* |
| *ATR* | *DIS3L2* | *GPS2* | *MAX* | *PRKAR1A* | *SOX9* |
| *ATRX* | *DNAJB1* | *GRB2* | *MCL1* | *PRKCE* | *SPEN* |
| *AURKA* | *DNMT1* | *GREM1* | *MDC1* | *PRKCG* | *SPINK1* |
| *AURKB* | *DNMT3A* | *GRIN2A* | *MDM2* | *PRKCI* | *SPOP* |
| *AXIN1* | *DNMT3B* | *GRM3* | *MDM4* | *PRKD1* | *SPRED1* |
| *AXIN2* | *DOT1L* | *GSK3B* | *MED12* | *PRKDC* | *SPTA1* |
| *AXL* | *DROSHA* | *GSTA1* | *MEF2B* | *PRRT2* | *SRC* |
| *B2M* | *DUSP4* | *H3F3A* | *MEN1* | *PRSS8* | *SRSF2* |
| *BABAM1* | *E2F1* | *H3F3B* | *MET* | *PTCH1* | *STAG2* |
| *BAP1* | *E2F3* | *H3F3C* | *MGA* | *PTCH2* | *STAT3* |
| *BARD1* | *EED* | *HDAC1* | *MITF* | *PTEN* | *STAT4* |
| *BAX* | *EGF* | *HDAC2* | *MLH1* | *PTK2* | *STAT5A* |
| *BBC3* | *EGFL7* | *HDAC3* | *MPL* | *PTP4A1* | *STAT5B* |
| *BCL10* | *EGFR* | *HDAC4* | *MRE11A* | *PTPN11* | *STK11* |
| *BCL11A* | *EIF1AX* | *HDAC6* | *MSH2* | *PTPRD* | *STK19* |
| *BCL2* | *EIF4A2* | *HDAC8* | *MSH3* | *PTPRS* | *STK40* |
| *BCL2L1* | *EIF4E* | *HGF* | *MSH6* | *PTPRT* | *SUFU* |
| *BCL2L11* | *ELF3* | *HIF1A* | *MSI1* | *QKI* | *SUZ12* |
| *BCL2L2* | *EML4* | *HIST1H1C* | *MSI2* | *RAB35* | *SYK* |
| *BCL6* | *EP300* | *HIST1H2BD* | *MST1* | *RAC1* | *TAF1* |
| *BCOR* | *EPAS1* | *HIST1H3A* | *MST1R* | *RAC2* | *TAP1* |
| *BCORL1* | *EPCAM* | *HIST1H3B* | *MTOR* | *RAD21* | *TAP2* |
| *BIRC3* | *EPHA3* | *HIST1H3C* | *MUTYH* | *RAD50* | *TBX3* |
| *BIRC5* | *EPHA5* | *HIST1H3D* | *MYC* | *RAD51* | *TCEB1* |
| *BLCAP* | *EPHA7* | *HIST1H3E* | *MYCL* | *RAD51B* | *TCF3* |
| *BLK* | *EPHB1* | *HIST1H3F* | *MYCN* | *RAD51C* | *TCF7L2* |
| *BLM* | *ERBB2* | *HIST1H3G* | *MYD88* | *RAD51D* | *TEK* |
| *BMPR1A* | *ERBB3* | *HIST1H3H* | *MYO1B* | *RAD52* | *TERT* |
| *BRAF* | *ERBB4* | *HIST1H3I* | *MYOD1* | *RAD54L* | *TET1* |
| *BRCA1* | *ERCC1* | *HIST1H3J* | *NAT1* | *RAF1* | *TET2* |
| *BRCA2* | *ERCC2* | *HIST2H3C* | *NAT2* | *RANBP2* | *TGFBR1* |
| *BRD3* | *ERCC3* | *HIST2H3D* | *NBN* | *RARA* | *TGFBR2* |
| *BRD4* | *ERCC4* | *HIST3H3* | *NCOA3* | *RARB* | *TMEM127* |
| *BRIP1* | *ERCC5* | *HLA-A* | *NCOR1* | *RASA1* | *TMPRSS2* |
| *BTG1* | *ERF* | *HLA-B* | *NEGR1* | *RASSF1* | *TNF* |
| *BTK* | *ERG* | *HNF1A* | *NF1* | *RASSF8* | *TNFAIP3* |
| *BUB1* | *ERRFI1* | *HOXB13* | *NF2* | *RB1* | *TNFRSF14* |
| *BUB1B* | *ESR1* | *HRAS* | *NFE2L2* | *RBM10* | *TNFSF11* |
| *BUB3* | *ETV1* | *HSD3B1* | *NFKBIA* | *RECQL* | *TOP1* |
| *C11orf30* | *ETV6* | *HSP90AA1* | *NKX2-1* | *RECQL4* | *TOP2A* |
| *CALR* | *EWSR1* | *ICOSLG* | *NKX3-1* | *REL* | *TP53* |
| *CARD11* | *EXT1* | *ID3* | *NOTCH1* | *RET* | *TP53BP1* |
| *CARM1* | *EXT2* | *IDH1* | *NOTCH2* | *RFWD2* | *TP63* |
| *CASP8* | *EZH1* | *IDH2* | *NOTCH3* | *RHBDF2* | *TPX2* |
| *CBFB* | *EZH2* | *IFNGR1* | *NOTCH4* | *RHEB* | *TRAF2* |
| *CBL* | *FAM175A* | *IGF1* | *NPM1* | *RHOA* | *TRAF7* |
| *CCND1* | *FAM46C* | *IGF1R* | *NRAS* | *RICTOR* | *TSC1* |
| *CCND2* | *FAM58A* | *IGF2* | *NRG1* | *RIT1* | *TSC2* |
| *CCND3* | *FANCA* | *IGF2R* | *NSD1* | *RNF43* | *TSHR* |
| *CCNE1* | *FANCB* | *IKBKE* | *NTHL1* | *ROCK1* | *TUBB* |
| *CD274* | *FANCC* | *IKZF1* | *NTRK1* | *ROS1* | *TWIST1* |
| *CD276* | *FANCD2* | *IL10* | *NTRK2* | *RPS6KA1* | *TYMS* |
| *CD74* | *FANCE* | *IL7R* | *NTRK3* | *RPS6KA4* | *U2AF1* |
| *CD79A* | *FANCF* | *IL8* | *NUF2* | *RPS6KB1* | *UPF1* |
| *CD79B* | *FANCG* | *INHA* | *NUP93* | *RPS6KB2* | *VEGFA* |
| *CDC25C* | *FANCI* | *INHBA* | *OPRM1* | *RPTOR* | *VEGFB* |
| *CDC42* | *FANCL* | *INPP4A* | *PAK1* | *RRAGC* | *VHL* |
| *CDC73* | *FANCM* | *INPP4B* | *PAK3* | *RRAS* | *VTCN1* |
| *CDH1* | *FAS* | *INPPL1* | *PAK7* | *RRAS2* | *WEE1* |
| *CDK1* | *FAT1* | *INSR* | *PALB2* | *RRM1* | *WHSC1* |
| *CDK12* | *FAT4* | *IRF2* | *PARK2* | *RTEL1* | *WHSC1L1* |
| *CDK2* | *FBXW7* | *IRF4* | *PARP1* | *RUNX1* | *WISP3* |
| *CDK4* | *FGF10* | *IRS1* | *PARP2* | *RUNX1T1* | *WNT1* |
| *CDK5* | *FGF14* | *IRS2* | *PARP3* | *RUNX2* | *WNT5A* |
| *CDK6* | *FGF19* | *JAK1* | *PAX5* | *RXRA* | *WNT6* |
| *CDK7* | *FGF23* | *JAK2* | *PBRM1* | *RYBP* | *WRN* |
| *CDK8* | *FGF3* | *JAK3* | *PDCD1* | *SDHA* | *WT1* |
| *CDK9* | *FGF4* | *JUN* | *PDCD1LG2* | *SDHAF2* | *WWTR1* |
| *CDKN1A* | *FGF6* | *KAT6A* | *PDGFRA* | *SDHB* | *XIAP* |
| *CDKN1B* | *FGFR1* | *KDM5A* | *PDGFRB* | *SDHC* | *XPA* |
| *CDKN1C* | *FGFR2* | *KDM5C* | *PDK1* | *SDHD* | *XPC* |
| *CDKN2A* | *FGFR3* | *KDM6A* | *PDPK1* | *SESN1* | *XPO1* |
| *CDKN2B* | *FGFR4* | *KDR* | *PEG3* | *SESN2* | *XRCC2* |
| *CDKN2C* | *FH* | *KEAP1* | *PGR* | *SESN3* | *YAP1* |
| *CEBPA* | *FLCN* | *KEL* | *PHOX2B* | *SETD2* | *YES1* |
| *CENPA* | *FLT1* | *KIT* | *PIK3C2B* | *SETD8* | *ZBTB2* |
| *CHD2* | *FLT3* | *KLF4* | *PIK3C2G* | *SF3B1* | *ZFHX3* |
| *CHD3* | *FLT4* | *KLHL6* | *PIK3C3* | *SH2B3* | *ZNF217* |
| *CHD4* | *FOLR3* | *KMT2A* | *PIK3CA* | *SH2D1A* | *ZNF703* |

**Supplementary Table 2.** Thirty-four DDR-related gene panel.

| **MMR** | **NER** | **HR** | **FA** | **Checkpoint** | **Others** |
| --- | --- | --- | --- | --- | --- |
| *MLH1* | *ERCC2* | *BRCA1* | *BRCA2* | *ATM* | *POLE* |
| *MSH2* | *ERCC3* | *MRE11A* | *BRIP1* | *ATR* | *MUTYH* |
| *MSH6* | *ERCC4* | *NBN* | *FANCA* | *CHEK1* | *PARP1* |
| *PMS1* | *ERCC5* | *RAD50* | *FANCC* | *CHEK2* | *RECQL4* |
| *PMS2* |  | *RAD51* | *PALB2* | *MDC1* |  |
|  |  | *RAD51B* | *RAD51C* |  |  |
|  |  | *RAD51D* | *BLM* |  |  |
|  |  | *RAD52* |  |  |  |
|  |  | *RAD54L* |  |  |  |

DDR: DNA damage repair; MMR: mismatch repair; NER: nucleotide excision repair; HR: homologous recombination, FA: fanconi anemia.

**Supplementary Table 3.** UTUC patient characteristics between Chinese and MSKCC cohort

| **Variables** | **Chinese (N = 131)** | **MSKCC (N = 119)** | **p-value** |
| --- | --- | --- | --- |
| **Diagnosis age** | 66 [36, 86] | 66 [38, 89] | 0.39 |
| **Gender** |  |  |  |
| Male | 79 | 78 | 0.43 |
| Female | 52 | 41 |  |
| **Smoker** |  |  |  |
| Yes | 20 | 0 | NA |
| No | 23 | 0 |  |
| NA | 88 | 119 |  |
| **Tumor site** |  |  |  |
| Renal pelvis | 74 | NA | NA |
| Ureter | 57 | NA |  |
| **Clinical stage** |  |  |  |
| Ⅰ/Ⅱ | 47 | NA | NA |
| Ⅲ/Ⅳ | 84 | NA |  |

UTUC: upper tract urothelial carcinoma; NA: not applicable.

**Supplementary Table 4.** UCB patient characteristics between Chinese and TCGA cohort

| **Variables** | **Chinese (N = 115)** | **TCGA (N = 413)** | **p-value** |
| --- | --- | --- | --- |
| **Diagnosis age** | 66 [19, 86] | 69 [34, 90] | **< 0.01** |
| **Gender** |  |  |  |
| Male | 90 | 305 | 0.40 |
| Female | 25 | 108 |  |
| **Smoker** |  |  |  |
| Yes | 16 | 225 | 0.51 |
| No | 25 | 188 |  |
| NA | 74 | 0 |  |
| **Muscle-invasive** |  |  |  |
| MIBC | 115 | 413 | 1.00 |
| **Clinical stage** |  |  |  |
| Ⅰ/Ⅱ | 31 | 0 | **< 0.01** |
| Ⅲ/Ⅳ | 84 | 411 |  |
| NA | 0 | 2 |  |

UCB: urothelial carcinoma of the bladder; MIBC: muscle-invasive bladder carcinoma; **Bold** represented there was a statistical significance; NA: not applicable.

**Supplementary Table 5.** UCB patient characteristics between Chinese and MSKCC cohort

| **Variables** | **Chinese (N = 115)** | **MSKCC (N = 80)** | **p-value** |
| --- | --- | --- | --- |
| **Diagnosis age** | 66 [19, 86] | 70 [45, 88] | **< 0.01** |
| **Gender** |  |  |  |
| Male | 90 | 59 | 0.50 |
| Female | 25 | 21 |  |
| **Smoker** |  |  |  |
| Yes | 16 | 55 | 0.42 |
| No | 25 | 25 |  |
| NA | 74 | 0 |  |
| **Muscle-invasive** |  |  |  |
| MIBC | 115 | 80 | 1.00 |
| **Clinical stage** |  |  |  |
| Ⅰ/Ⅱ | 31 | 0 | NA |
| Ⅲ/Ⅳ | 84 | 0 |  |
| NA | 0 | 80 |  |

UCB: urothelial carcinoma of the bladder; MIBC: muscle-invasive bladder carcinoma; **Bold** represented there was a statistical significance; NA: not applicable.

**Supplementary Table 6.** A shared panel of 209 genes between Chinese and MSKCC cohorts

| *ABL1* | *CHEK2* | *FBXW7* | *MRE11A* | *NOTCH2* | *PTPRS* |
| --- | --- | --- | --- | --- | --- |
| *ABL2* | *CREBBP* | *FGFR1* | *NBN* | *NOTCH3* | *RAF1* |
| *AKT1* | *CRKL* | *FGFR2* | *PMS2* | *NPM1* | *RARA* |
| *AKT2* | *CSF1R* | *FGFR3* | *KDM5C* | *PPP2R1A* | *RB1* |
| *AKT3* | *CDK12* | *FGFR4* | *KDM6A* | *PTPRT* | *REL* |
| *ALK* | *CDKN1A* | *FH* | *KDR* | *RNF43* | *RET* |
| *ALOX12B* | *CIC* | *FLCN* | *KEAP1* | *RAD50* | *RICTOR* |
| *APC* | *E2F3* | *FLT1* | *KIT* | *RPS6KB1* | *RPTOR* |
| *AR* | *CTCF* | *FLT3* | *KMT2A* | *NTRK1* | *SDHB* |
| *ARAF* | *ERCC2* | *GATA1* | *KMT2D* | *NTRK2* | *SETD2* |
| *ARID1A* | *CTNNB1* | *GATA3* | *KMT2C* | *NTRK3* | *SMAD4* |
| *ASXL1* | *CYLD* | *GNAQ* | *KRAS* | *PARP1* | *SMARCA4* |
| *ATM* | *DDR2* | *FLT4* | *MAGI2* | *PAX5* | *SMARCB1* |
| *ATRX* | *DICER1* | *FUBP1* | *MAP2K2* | *PBRM1* | *SMO* |
| *AURKA* | *DIS3* | *GLI1* | *MEN1* | *PDGFRA* | *U2AF1* |
| *BAP1* | *DNMT1* | *SMAD3* | *MET* | *PDGFRB* | *XPO1* |
| *BCL6* | *DNMT3A* | *IRF4* | *MITF* | *PHOX2B* | *SPOP* |
| *BRAF* | *EGFR* | *MAPK1* | *MED12* | *PIK3C2G* | *SRC* |
| *BRCA1* | *EP300* | *GNAS* | *MEF2B* | *PIK3CA* | *STK11* |
| *BRCA2* | *EPHA3* | *GRIN2A* | *MYD88* | *PIK3CB* | *TEK* |
| *ARID2* | *EPHA5* | *GSK3B* | *PALB2* | *PIK3CD* | *TERT* |
| *BCL2L11* | *EPHB1* | *HDAC2* | *POLE* | *PIK3R1* | *TET1* |
| *BCOR* | *ERBB2* | *HNF1A* | *PPP6C* | *PIK3R2* | *TET2* |
| *BUB1B* | *ERBB3* | *HRAS* | *MLH1* | *PIK3R3* | *TGFBR2* |
| *ARID1B* | *ERBB4* | *HSP90AA1* | *MPL* | *PLK2* | *TMPRSS2* |
| *ATR* | *FAT1* | *IDH1* | *MSH2* | *PNRC1* | *TP53* |
| *CCND2* | *SMAD2* | *IGF1R* | *MSH6* | *PREX2* | *TP63* |
| *CCNE1* | *ERCC5* | *IKZF1* | *MTOR* | *ROS1* | *TSC1* |
| *CDC73* | *FAT4* | *INSR* | *MYC* | *SF3B1* | *TSC2* |
| *CDH1* | *ERG* | *IRS2* | *MYCN* | *SRSF2* | *TSHR* |
| *CDK4* | *ESR1* | *JAK1* | *NF1* | *STAG2* | *VHL* |
| *CDK6* | *ETV1* | *JAK2* | *NF2* | *PRKCI* | *WT1* |
| *CDKN2A* | *EZH2* | *JAK3* | *NFE2L2* | *PTCH1* | *YAP1* |
| *CEBPA* | *FAM46C* | *IL7R* | *NKX2-1* | *PTEN* | *YES1* |
| *CHEK1* | *FAS* | *INPP4A* | *NOTCH1* | *PTPRD* |  |
